# Applications of Ferric Oxide in Water Splitting by Electrolysis: A Comprehensive Review

**DOI:** 10.3390/molecules29214990

**Published:** 2024-10-22

**Authors:** Bruno G. Pollet, Shankara S. Kalanur

**Affiliations:** Green Hydrogen Lab (GH2Lab), Hydrogen Research Institute (HRI), Université du Québec à Trois Rivières (UQTR), 3351 Boulevard des Forges, Trois-Rivières, QC G9A 5H7, Canada; bruno.pollet@uqtr.ca

**Keywords:** water splitting, Fe_2_O_3_, bifunctional catalyst, oxygen evolution reaction, hydrogen

## Abstract

In water electrolysis, the use of an efficient catalyst derived from earth-abundant materials which is cost-effective and stable is essential for the economic sustainability of hydrogen production. A wide range of catalytic materials have been reported upon so far, among which Fe_2_O_3_ stands out as one of the most credible candidates in terms of cost and abundance. However, Fe_2_O_3_ faces several limitations due to its poor charge transfer properties and catalytic ability; thus, significant modifications are essential for its effective utilization. Considering the future of water electrolysis, this review provides a detailed summary of Fe_2_O_3_ materials employed in electrolytic applications with a focus on critically assessing the key electrode modifications that are essential for the materials’ utilization as efficient electrocatalysts. With this in mind, Fe_2_O_3_ was implemented in a heterojunction/composite, doped, carbon supported, crystal facet tuned system, as well as in metal organic framework (MOF) systems. Furthermore, Fe_2_O_3_ was utilized in alkaline, seawater, anion exchange membrane, and solid oxide electrolysis systems. Recently, magnetic field-assisted water electrolysis has also been explored. This comprehensive review highlights the fact that the applicability of Fe_2_O_3_ in electrolysis is limited, and hence, intense and strategically focused research is vital for converting Fe_2_O_3_ into a commercially viable, cost-effective, and efficient catalyst material.

## 1. Introduction

The energy derived from fossil fuels was the main driving force for the industrial and economic growth of the 19th century [1]. At present, roughly 60 to 80 percent of the total energy produced and consumed in developed countries is derived from fossil fuels such as coal, oil, and natural gas, supporting infrastructure systems such as electricity generation and transportation, as well as industry [2]. The past century shows the importance of fossil fuels and energy sources regarding the navigation of human advancement through industrialization and economic growth. However, the extensive usage of fossil fuels poses serious concerns in terms of greenhouse gases (GHG) and pollution, which cause an imbalance in nature, leading to environmental calamities [3]. Moreover, fossil fuels are non-renewable and geopolitically controlled natural resources which pose challenges in terms of constant extraction and controlled supply chains; thus, their replacement is inevitable [4]. Importantly, a profound shift toward carbon-free, renewable, and sustainable energy resources is essential in terms of safeguarding the environment and supporting economic stability along with industrial growth. Hence, the current focus of research presents an unprecedented opportunity to regulate global warming via the development of carbon-neutral technologies.

Among the currently available green (or renewable) energy contenders, hydrogen has been found to be the ideal option to replace fossil fuels [5]. The abundance of atomic and molecular hydrogen on Earth in a wide range of naturally available molecules such as water, fossil fuels, etc. makes it a potential candidate as a future renewable energy source [6]. The superiority of hydrogen as a source of energy compared with other sources is due to its extensive utilization in wider applications that provide energy (heat/electricity) with a high specific energy density [7] and to the fact that it releases carbon-free water as a byproduct, e.g., when used in fuel cells [8,9,10]. Hence, the demand for hydrogen is significantly increasing and is expected to continue to rise in the coming decades owing to industrial applications in addition to applications in energy infrastructure. The availability and green production of hydrogen in all parts of the globe (water), combined with sustainable routes of extraction, make it an ideal energy source for energy independence, regardless of geopolitical situations. Given its advantages and demand, the extensive research advancements in hydrogen-driven energy and industrial utilization could experience exponential growth due to the commitment of major economic players to shift toward carbon-free technologies.

**Figure 1:**
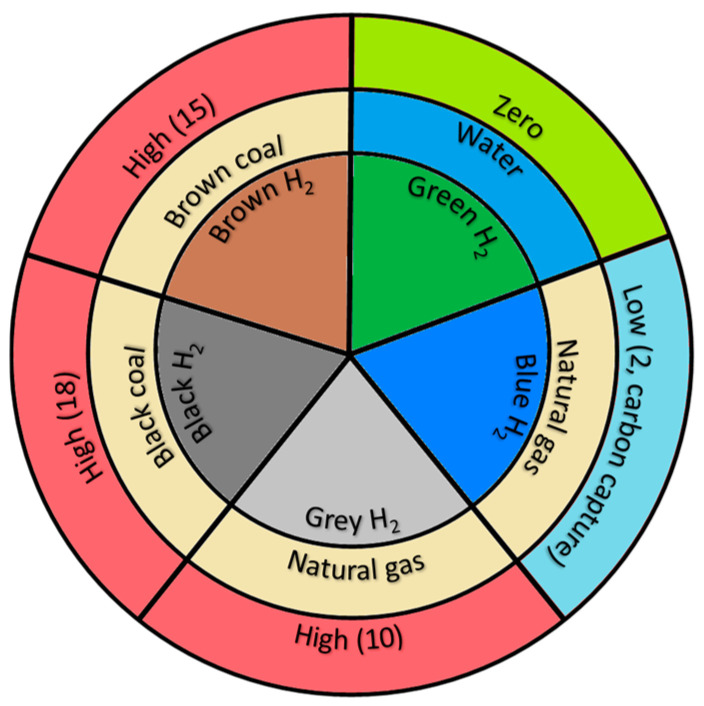
Types of hydrogen (green, blue, grey, black, and brown hydrogen) produced (first circle) around the globe, along with their sources (second circle) and their carbon emission levels (third circle). Approximate values of kgCO_2_/kgH_2_ produced are indicated.

Advancement toward a hydrogen infrastructure has gained significant attention and funding recently, as it plays an important role in achieving the Paris Agreement target set for the reduction in emissions by 2050 [11]. To achieve this target, reducing the cost of hydrogen is crucial; this depends on hydrogen production technologies, storage, transportation, and utilization. Recent developments reveal that the rapid and onsite/remote availability of hydrogen from a wide range of production technologies is capable of significantly reducing storage and transportation expenses. However, the choice of suitable hydrogen production technologies is a vital factor that influences the overall cost of hydrogen (LCOH—levelized cost of hydrogen). Figure 1 represents the various types of hydrogen produced around the globe, along with their sources of production and the amount of carbon emissions associated with these production technologies. Note that, except for green hydrogen, other methods of hydrogen production (black, blue, brown, and grey) use fossil fuels as a source of hydrogen; only green hydrogen uses water as a source (if renewables and water electrolysis are used). Moreover, except for blue and green hydrogen technologies, all other methodologies emit enormous amounts of GHG and are thus not recommended as sources of energy in the future. Notably, the reduction in carbon emissions (such as carbon dioxide, CO_2_) for blue hydrogen is due to the carbon capture and sequestration (CCS) processes linked to those hydrogen production systems.

Among large-scale hydrogen production technologies, the steam methane reforming (SMR) method dominates as the most cost-effective production technology (>99%) [12]. However, the utilization of fossil fuels in SMR leads to enormous carbon emissions. Even though SMR technology could be converted to blue hydrogen by combining it with CCS, its dependence on fossil fuels makes it a geopolitical challenge and expensive (due to carbon capture processing) compared to the other technologies. Therefore, the use of carbon-free sources and methods, along with the use of renewable energy, is essential for achieving cost-effective, pilot-scale hydrogen production in order to fulfil the emission target and achieve cost reductions. In particular, rigorous research and development are essential to explore, develop, and exploit greener hydrogen production technologies that could meet future demands and are cost effective.

## 2. Overview of Electrochemical Water Splitting

Electrochemical water splitting is a process of water oxidation and reduction to produce O_2_ and H_2_ under an applied potential bias/electricity, as shown in Equation (1). The phenomenon of the decomposition of molecules using an applied current was first observed in 1789 and was later applied to in water electrolysis in 1800 by Nicholson and Carlisle [13]. Later, water electrolysis methodology was optimized and further developed into a mature technology for increased efficiency and practicality. Recently, the importance of water electrolysis has gained significant attention, given that it is carbon-free approach to hydrogen production utilizing renewable and sustainable resources. For electrolysis, the water required for H_2_ and O_2_ production could be sourced from the sea or any water body, and electricity generated using green and inexpensive renewable sources, such as solar and wind, could provide the required electrical energy to split the water molecules. Thermodynamically, the energy required to split water into H_2_ and O_2_ is shown in the below equation.

H_2_O + Electricity (237.2 kJ mol^−1^) + Heat (48.6 kJ mol^−1^) → H_2_ + ½ O_2_ (1)

**Figure 2:**
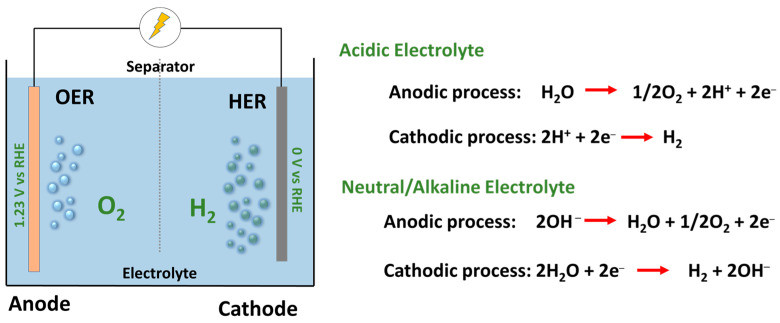
Schematic presentation of electrochemical water splitting (electrolysis) under acidic, neutral, and alkaline conditions.

The above equation represents an ideal condition required in terms of the energy, electrodes, and charge dynamics required to split water into H_2_ and O_2_. Electrochemically (thermodynamic cell voltage), a minimum bias voltage of 1.229 V vs. a standard hydrogen electrode (SHE) is required to oxidize water at the anode to produce O_2_ and to produce H_2_ gas at the cathode surface. However, under normal experimental conditions, the required voltage has been found to be higher than the theoretical values (>1.229 V vs. SHE) to overcome the ohmic resistance of the electrolyte and the reaction kinetics in the electrochemical cell. The process of water electrolysis in an electrochemical cell is shown in Figure 2. To split water, the electrode materials are connected to the anode and cathode supply of the power output and are immersed in a suitable electrolyte. Under the applied electricity, the water molecules are split into O_2_ via oxygen evolution reaction (OER) and H_2_ through hydrogen evolution reaction (HER) at the anode and cathode surfaces, respectively. Generally, the anode and cathode compartments are separated using a separator/diaphragm/membrane to avoid the mixing of H_2_ and O_2_ gases and to maintain the flow of ions between the compartments.

Water electrolysis can be achieved in a wide range of electrolytes of different pH values with the utilization of efficient and stable electrodes. In acidic electrolytes, the water is oxidized at the anode surface, which gives two electrons, whereas the H^+^ ions are diffused through the separator/membrane toward the cathode to combine with electrons to yield H_2_ gas. In neutral and alkaline electrolytes, OH^−^ ions are consumed to produce O_2_ gas along with two electrons and a water molecule, which are reduced to H_2_ at the cathode surface with the release of OH^−^ ions. The constant production of H_2_ and O_2_ is balanced by the production and diffusion of the relevant ions toward the respective electrode surface (Figure 2). In water electrolysis, the use of electrolytes is essential to provide significant conductivity between the electrodes, i.e., to decrease the ohmic resistance and create a feasible ionic environment at the electrode surface for effective OER and HER activity. Hence, the utilization of electrolytes with high ionic mobility is essential to decrease the thermodynamic barrier and energy requirement to oxidize and reduce water molecules. Generally, potassium hydroxide (KOH), sulfuric acid (H_2_SO_4_), sodium sulfate (Na_2_SO_4_), phosphate buffers, etc. are used as electrolytes in the water electrolysis systems.

## 3. Electrolysis Techniques

**Figure 3:**
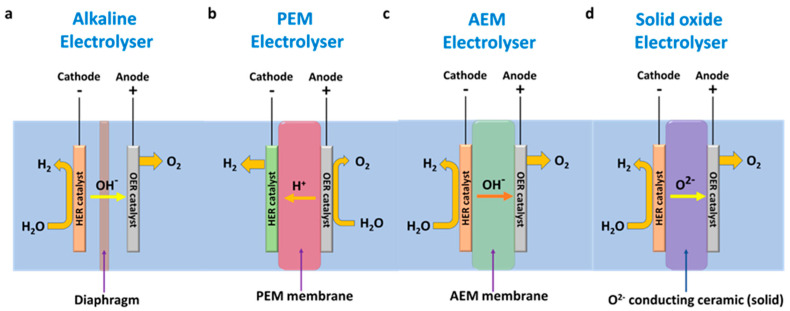
Schematic presentation of different types of electrolyzers: (**a**) alkaline water electrolyzers, (**b**) PEM electrolyzers, (**c**) AEM electrolyzers, and (**d**) solid oxide electrolyzers.

Electrolysis techniques are generally classified based on the setup and components utilized, as shown in Figure 3, among which alkaline water electrolysis (AWE), proton exchange membrane water electrolysis (PEMWE), anion exchange membrane water electrolysis (AEMWE), and solid oxide electrolysis cells (SOECs) are the most widely used methods (Figure 3). In AWE, the anode and cathode half-cell reactions take place (under the applied cell potentials) in different compartments separated by diaphragms/separators that allow the effective diffusion of OH^−^ ions from the cathode (after HER) to the anode side to carry out the OER process. AWE is generally operated with very high pH electrolytes (up to 5–7 M) using Ni-based electrodes. Due to its simplistic design and minimal component requirements, AWE is widely used in hydrogen production at pilot/industrial scales. In PEMWE, the anode and cathode sides are separated by a PEM (usually Nafion^®^), which facilitates the effective diffusion of H^+^ ions. During the electrolysis, the water molecules are oxidized at the anode side, producing H^+^ ions that are transferred to the cathode side via the PEM for the water reduction reaction. For the electrolysis, acidic electrolytes are passed through the anode side containing an OER (iridium dioxide, IrO_2_) catalyst. At the cathode side, platinum (Pt) nanoparticle-supported on carbon black (Pt/C) catalysts are widely employed as electrodes for HER processes. The PEM setup involves the use of gas diffusion layers (GDL), which yield high-purity H_2_ compared to that produced using AWE.

The AEMWE was developed by combining the advantages of AWE and PEMWE. The AEMWE is a membrane-based alkaline electrolyzer that produces high-purity H_2_ in mild basic conditions without the use of platinum group metal (PGM) catalysts. During electrolysis, the basic electrolyte is fed into both the anode and cathode side to produce H_2_, whereas the OH^−^ ions diffuse toward the anode through the AEM to generate O_2_. Recently, AEMWE has demonstrated promising breakthroughs, indicating its commercial viability and pilot-scale production capability. SOEC systems are operated at very high temperatures (>700 °C) to split water into O_2_ and H_2_. At elevated temperatures, the water is fed in the form of steam at the cathode side, where H_2_ is produced. A solid ceramic membrane was used to separate the anode and cathode sides, allowing the effective diffusion of O^2–^ ions from the cathode to the anode cell. Similar to AEMWE, SOEC has exhibited promising commercial viability, indicating its potential contribution to hydrogen production in the future.

In water electrolysis systems, the catalysts and electrodes employed for OER and HER activities play a vital role in determining the overall efficiency, stability, and long-term operation potential. Noble metals (PGMs) are widely known for their efficient catalytic activity toward OER and HER and thus are routinely used in commercial and pilot-scale electrolyzers. In particular, the noble metals show significantly low overpotential (*η*) to split water into H_2_ and O_2_ in comparison to the non-noble metal catalysts. Hence, a non-noble metal catalyst needs comparatively higher overpotentials to perform OER and HER. Despite the efficiency factor, the cost, abundance, and geographical conditions need to be considered for commercial acceptance, along with cost reductions. Hence, catalysts derived from non-noble metals are recommended, as these are cost-effective, abundant, and stable. With this in mind, the catalysts/electrodes play a crucial role in determining the hydrogen production cost, and thus, the viability of commercial hydrogen. However, non-noble catalysts show limited catalytic activity, and thus, various modifications and different strategies are essential [14,15,16] for their effective implementation in electrolyzers. Therefore, the choice of a suitable catalyst and the required modifications are critical in developing efficient catalysts for water splitting in electrolyzers.

## 4. Fe_2_O_3_

The use of metal oxides in OER processes is known to be an effective approach, as the surface of metal oxides (MOx) provides essential sites for reaction intermediates to be adsorbed to initiate OER activity. Specifically, the adsorption and desorption of essential reaction intermediates on the metal oxide surface is an important step toward achieving water splitting reactions. Reaction intermediates with optimum adsorption energies at the oxide surface hold the key to improving water splitting efficiency [17]. Moreover, the conductive nature of MOx means that it serves as an effective charge diffusion/transfer medium. MOx can be obtained with different nanostructures, crystal phase/planes, and doped, and it can be anchored to a wide range of catalysts/substrates. Hence, the use of MOx in electrocatalytic processes offers a promising strategy for hydrogen production. Among metal oxides, Fe_2_O_3_ is an abundant mineral form of Fe metal (in addition to other minerals) in nature. The hematite mineral form of Fe_2_O_3_ is the naturally occurring form of iron, a known primary element extracted from the earth’s crust. In general, Fe_2_O_3_ has a dark red or brown color and has various polymorphs. Fe_2_O_3_ is known for its relatively weak ferromagnetic properties at room temperature; it also exhibits antiferromagnetic properties below 260 K. Even though the magnetic properties of Fe_2_O_3_ are not known to directly influence its other applications, they have been found to affect its carrier transport properties and optoelectronic characterizations, owing to the various spin configurations. Furthermore, Fe_2_O_3_ exhibits strong photon absorption in the visible yellow region to UV, with a photon transmission in the orange to the infrared region that results in the typical red appearance. Fe_2_O_3_ has a band gap of 2.1 eV, which accounts for the absorption of ~40% of sunlight and thus presents semiconductor properties. Fe_2_O_3_ is highly stable and can be easily dissolved/reacted with most acids. The other forms of iron oxides generally found in nature are FeO (iron (II) oxide) and Fe_3_O_4_ (magnetite). Due to its abundance, cost, absorbance, stability, and non-toxic nature, Fe_2_O_3_ is effectively utilized in a wide range of applications [18], including as an anode material in batteries [19], in supercapacitors [20], in gas-sensing [21], in electrochemical sensors [22], for photoelectrochemical water splitting [23], dye degradation [24], in biomedical applications [25], and in electrolysis [26]. In particular, considering its OER capabilities under the applied bias potential (with or without illumination), Fe_2_O_3_-based electrodes have been used for water-splitting applications using both photoelectrochemical [27] and electrocatalytic modes [28]. Furthermore, Fe_2_O_3_ has been effectively employed in biomedical applications due to its superior permeability and stability. In particular, Fe_2_O_3_ has been utilized to transfer therapeutic agents through tissues and in controlled drug-releasing agents. Due to its non-toxic nature, Fe_2_O_3_ is well suited as nano-carriers for drugs that could limit and regulate the drug dosage and supply, resulting in more effective treatments. The utilization of Fe_2_O_3_ in medicine offers effective treatments for drug release and limits overdosages to certain body sites. Recent work on Fe_2_O_3_ applications has led to Fe_2_O_3_ being recognized as a superior catalyst molecule when employed directly and as a support material. Owing to its unique physiochemical properties, two different valence oxidation states, and surface properties, Fe_2_O_3_ is being effectively employed in energy applications. Therefore, the use of Fe_2_O_3_-based materials as electrodes, photoelectrodes, and catalysts could provide a promising opportunity for cheap hydrogen production technologies.

Fe_2_O_3_ is found in α-, β-, γ-, and ε- polymorphs, having different properties and stabilities. Among the polymorphs, the α phase has been widely studied owing to its stability and feasible application in charge storage devices and as pigments in paints [29,30]. β-Fe_2_O_3_ contains a bixbyite structure and is a rarely studied polymorph due to its intermediary metastable condition, which transforms to α-Fe_2_O_3_ at higher temperatures [31]. The γ polymorph of Fe_2_O_3_, also known as maghemite, contains a cubic structure and shows ferromagnetic properties [32]. The ε-Fe_2_O_3_ polymorph is a well-known magnetic material exhibiting a non-centrosymmetric orthorhombic structure [33]. Among the polymorphs, both α and γ show interchangeable crystalline forms, in which α-Fe_2_O_3_ becomes dominant and stable at higher temperatures when treated in an inert atmosphere and with a reducing agent [34].

**Figure 4:**
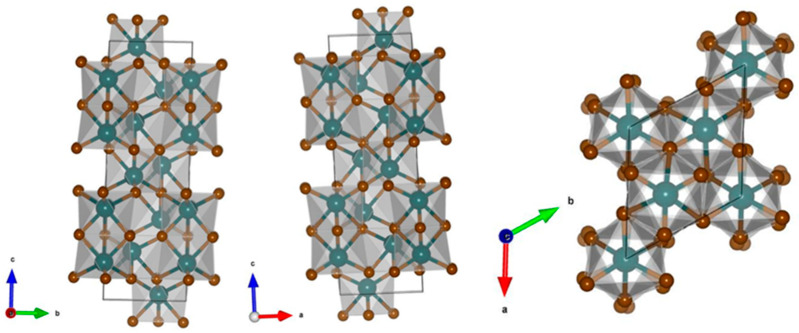
Crystal structure of α-Fe_2_O_3_ having a rhombohedral system of $\overset{-}{R}$3c space groups, showing a trigonal-hexagonal scalenohedral geometry.

Hematite (Fe_2_O_3_) is commonly described as having a hexagonal crystal structure. However, the fundamental crystal system is found to have a rhombohedral structure, that is, a hexagonal unit cell with specific parameters is used to describe the rhombohedral lattice of hematite Fe_2_O_3_. A typical crystal structure of hematite Fe_2_O_3_ is presented in Figure 4 (generated using Vesta [35]). The rhombohedral crystal system of hematite shows $\overset{-}{R}$3c space groups which could be transformed and described using hexagonal coordinates. Note that even though the underlying crystal structure of hematite Fe_2_O_3_ is rhombohedral, it is often described in terms of a hexagonal lattice system for convenience, as shown in Figure 4. In hematite α-Fe_2_O_3_ having $\overset{-}{R}$3c space group, six equivalent O^2−^ atoms are bonded to one Fe^3+^ atom to form FeO_6_ octahedra, whereas the lattice O^2−^ ions appear to be bonded to four equivalent Fe^3+^ atoms. The O^2−^ atoms bonded to Fe^3+^ form a mixture of distorted edge and corner-sharing OFe_4_ trigonal pyramids, as shown in Figure 4, whereas the FeO_6_ octahedra is found to form an edge and corner-sharing, along with a distorted face configuration with the neighboring octahedra.

### 4.1. Water Splitting Applications of Fe_2_O_3_

**Figure 5:**
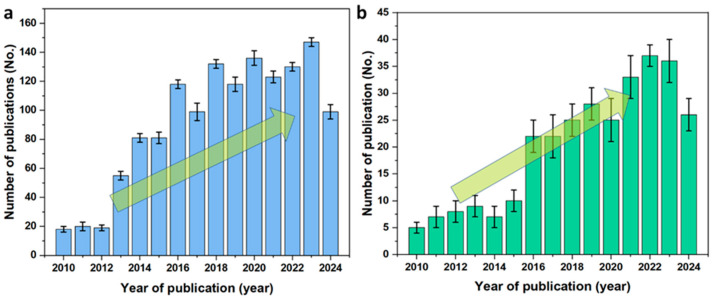
Articles on Fe_2_O_3_ for (**a**) water splitting (including solar, electrochemical, and photoelectrochemical routes) and (**b**) electrocatalytic water splitting.

Recently, Fe_2_O_3_ has been extensively used in water-splitting applications in both photoelectrochemical and electro-catalytical modes. Among the widely explored transition metals, Fe demonstrates considerably superior electrocatalytic activity, including HER, OER, and the oxygen reduction reaction (ORR), even compared to widely used Co and Ni-based materials [36,37,38]. Owing to its valency between the (III) and (II) states that offers exceptional catalytic properties and stability in comparison with the Ni and Co-based metal oxides, Fe possesses a feasible reversible redox capability [39]. Figure 5a shows the number of research articles published (photoelectrochemical and electrocatalytic modes) on the use of Fe_2_O_3_ electrodes for water splitting in recent decades. A gradual increase in the number of articles indicates that Fe_2_O_3_ is gaining significant attention owing to its semiconductor and catalytic properties. The extensive utilization of Fe_2_O_3_ in photoelectrochemical and photocatalytic applications is evident, owing to its absorbance and band edge properties that facilitate OER activity using photogenerated charges. Figure 5b shows the number of articles published during the last decade concerning the electrocatalytic water-splitting activity of Fe_2_O_3_, indicating the importance of Fe_2_O_3_ in water-splitting applications. In electrocatalytic water splitting systems, Fe_2_O_3_ materials with relevant modifications, including heterojunction with oxides/carbon/hydroxide/MOF systems, doping, oxygen vacancy tuning, etc., have been employed. In addition, Fe_2_O_3_ has exhibited significant activity as a catalyst in AEM and SOEC systems. Table 1 shows the literature reports on Fe_2_O_3_ in electrocatalytic water splitting applications. Additionally, the sections below provide a detailed discussion of various catalytic activity and electrochemical characterizations.

### 4.2. DFT Studies on the OER Activity of Fe_2_O_3_

Density functional theory (DFT) calculations offer a powerful tool to provide significant information regarding the surface and intrinsic properties of different elements/materials. In the case of Fe_2_O_3_, different surface orientations/crystal planes exhibit different atomic arrangements, distance defects, and atom densities which, in turn, affect their electronic structure and reactivity. Specifically, the adsorption of OER intermediates, their catalytic nature, and the stability of different facets/planes demonstrate important parameters in determining the efficiency of the water splitting. With this in mind, a wide range of DFT studies have been reported, highlighting the effects of different facets and their OER activities. Mainly, the (0001) facet of Fe_2_O_3_ has been reported to be more favorable for OER compared to other facets. The OER activity on the (0001) facet of completely hydroxylated Fe_2_O_3_ favors [73] the effective adsorption of reaction intermediates, resulting in enhanced OER activity. Furthermore, the doping of Fe_2_O_3_ (containing a fully hydroxylated surface) at the Fe and O sites using metal ions and anions, respectively, shows improved activity. For example, Ni and Co doping in Fe_2_O_3_ lead to the formation of a thermodynamically favored surface for effective adsorption reaction intermediates and increased efficiency of OER activity, resulting in low overpotentials. In-depth studies have indicated that active anion dopants at O lattice sites could improve the stability of the holes [73]. Furthermore, the surface states present at the (0001) facet of Fe_2_O_3_ have been found to affect the OER activity. The formation of a stable peroxo Fe-O-O-Fe adsorbate at the Fe_2_O_3_ surface has been found to be possible, whereas the presence of a Fe-O∙ type bond could be effective and is one of the possible intermediate pathways for the OER activity at the (0001) surface [74].

Similarly, DFT studies performed on the different exposed facets of Fe_2_O_3_ [75] have indicated a specific favorable OER orientation. Several facets of Fe_2_O_3_ have been studied, including (211), (021), (101), (210), and (100), which have, in general, been found to be less dominant in terms of OER activity. The calculations were performed by considering the free energy and overpotential values that dictate the four electron-coupled electron transfer reactions with OER intermediates. Studies revealed that the different orientations showed different free energy and overpotential values owing to the activity of intermediate species adsorbing on the facets, resulting in varied stability concerning the free energy values, which affects the resulting OER performance. Specifically, the orientation of Fe_2_O_3_ along (100) has been found to show relatively decreased overpotential values compared to the (211), (021), (101), and (210) orientations. Notably, the surface site in Fe_2_O_3_ that is responsible for the bridging of two Fe atoms on the (100) surface is favorable for adsorbate interactions, leading the enhanced OER activity. Interestingly, the (110) facet of Fe_2_O_3_ is known to be one of the favorable orientations for OER activity, as demonstrated by Zhang et al. [76] because, at the (100) facet, a noticeable competing OER reaction intermediate was observed at a single site between the O-O coupling and the formation of OOH. Here, O–O coupling refers to the involvement of two adjacent terminal Os at a dual site, leading to O–O bond formation. Furthermore, the presence of oxygen vacancies plays a critical role in decreasing the OER overpotentials [76]. Conclusively, DFT studies have indicated that the possible reaction pathways and favorable facets of Fe_2_O_3_ could be modified via the surface state, vacancies, and doping.

### 4.3. Crystal Facet and Morphology Tuning in Fe_2_O_3_

The morphology and the exposed crystal plane of Fe_2_O_3_ have a significant influence on the OER activity [77]. Generally, the interpretation of the structure−activity of electrodes/catalysts is cumbersome when analyzed using conventional electrochemical techniques. In most cases, interpretations of the results have been based on the average activity rather than a specific site or plane of activity, yielding an unambiguous conclusion. Therefore, for better clarity and more accurate interpretations, the direct mapping of electrochemical activities on the isolated crystals is essential. Using scanning electrochemical cell microscopy, Li et al. [77] proposed a single-entity electrochemical strategy to investigate the site-specific OER activity of Fe_2_O_3_. In their study, a single nanorod crystal of Fe_2_O_3_ was considered to monitor the OER activity; heterogeneity between and within the single nanorod crystal was observed. The single crystal Fe_2_O_3_ nanorods were obtained using the hydrothermal method. The produced nanorods has 0.5–1.5 μm rod lengths with a width of ~150 nm. The high-resolution morphology study confirmed significant electrochemical activity owing to the {001} plane present in the nanorod body. Accordingly, the enhanced OER activity was recorded at the {100} facet at the tip, which exhibited significantly increased activity. Hence the facet-dependent OER activity indicated that producing longer Fe_2_O_3_ nanorods with desired exposed facets would be beneficial compared to the other morphologies. Importantly, the proposed single-entity and sub-entity mapping strategy were shown to be an effective route to explore the intrinsic, surface, and facet-dependent catalytic activity; this approach could be broadly extended to other metal oxide nanostructures. The most important aspect of that study was the interpretation of the structural characteristics of most reactive OER sites on the surface of Fe_2_O_3_, which is crucial to optimize and engineer catalytic properties which could be applied to electrolyzers. Within the framework of a nanostructure, a crystal plane with active reactive sites is the key that needs to be recognized by engineering a specific nanoarchitecture which is generally hidden, shadowed, or removed/decreased in scale due to the presence of other nonreactive sites/planes.

### 4.4. Doped Fe_2_O_3_ Catalysts

The properties of metal oxides can be tuned/engineered to enhance electrochemical properties by introducing cations/anions into the crystal via doping [78]. In the case of Fe_2_O_3_, the intrinsic and surface properties could be altered/modified by doping. Generally, doping is achieved using a cation or an anion (or both) that displaces Fe or O atoms in the lattice. Doping could be performed during the synthesis process or in subsequent steps at high temperatures that ensure the effective displacement of Fe or O atoms from their lattice locations. The strategy of doping has advantages for materials like Fe_2_O_3_, which are abundant and cheap materials that show poor catalytic properties in their base form. Based on the nature of the dopant material, the properties of Fe_2_O_3_ could be tuned/engineered for optimum efficiency. For example, the utilization of Ni or Zn as a redox and non-redox active species, respectively, has been found to have a significant influence on the catalytic activity of Fe_2_O_3_, as noted by Shah et al [40]. Pristine and doped (Ni and Zn) Fe_2_O_3_ were obtained using a flame combustion method, followed by ball milling at 25 Hz for 15 min. Even though Ni is known to be effective as a dopant, Zn doping in Fe_2_O_3_ yielded significantly higher electrocatalytic properties. A low overpotential value of 350 mV was observed to reach 10 mA cm^−2^. The experimental and theoretical calculations indicated that the Ni dopant sites had a redox characteristic, whereas the Zn dopant sites on Fe_2_O_3_ showed a non-redox characteristic. With this in mind, Ni tends to lower the energetic barrier of the OER process, resulting in improved catalytic activity compared to pristine samples. However, in the case of Zn doping, a unique reaction pathway was observed, which is termed as proton-coupled electron transfer; this approach is thermodynamically favored and thus showed enhanced OER activity. Pristine F_2_O_3_ offers routine four-electron oxidation reaction sites, while Zn-Fe_2_O_3_ provides a rapid and effective reaction site that utilizes efficient two-site reaction pathways. Here, the Zn site was shown to possess a non-redox characteristic, as it maintained a constant charge during the water oxidation process.

Based on the nature of the dopant, doping amount, and site, a significant alteration of the surface and bulk properties of Fe_2_O_3_ could be achieved. For example, the doping of Se in Fe_2_O_3_ linked to Ni/NiO particles was reported to show efficient OER activity [41]. The synthesis of Se-doped Fe_2_O_3_ on a Ni/NiO nanostructure was achieved using a thermal method that supported in situ deposition directly on carbon fibers. The formation of a solid solution was not observed in the proposed method due to the limited etching of Fe to the carbon support, leading to the doping of Se in Fe_2_O_3_ that inhibited the diffusion to Ni system to form Se-Fe_2_O_3_-Ni/NiO. This characterization indicated that the employed technique successfully incorporated nanoparticles of Se-Fe_2_O_3_-Ni/NiO into the porous structure of the carbon fiber. LSV measurements performed in 1M KOH indicated the production of 10 mA cm^−2^ current density at an impressively lower overpotential value of 205 mV for the OER. Furthermore, a low Tafel slope of 36 mV dec^−1^ was recorded, with a low charge transfer resistance at the electrolyte interface. The superior conducting property of the carbon substrate and the systematic arrangement of Fe_2_O_3_@Ni/NiO nano junction systems in the porous carbon substrate structure, along with favorable interfaces (Se-Ni/NiO and Se-Fe_2_O_3_), led to an impressive electrochemical result, confirming the efficient OER activity of the Se-Fe_2_O_3_-Ni/NiO electrode.

The introduction of nonmetal/anion dopants into a Fe_2_O_3_ lattice is known to improve its electrocatalytic properties [79]. Unlike doping with Se, doping with P transforms Fe_2_O_3_ into an efficient bifunctional catalyst which has been utilized only as an OER catalyst [42]. Previous studies have shown that the introduction of PO_4_ into Fe_2_O_3_ improves the catalytic activity; this could be synthesized by performing electrochemical cycles in phosphate buffers [80]. The doping of P in iron oxides is known to regulate the electrical properties [79,81] which, in turn, effects the electrocatalytic efficiency. Generally, a support material is required for Fe_2_O_3_ owing to its limitation regarding the mass transfer process. In such a case, different metal oxides and morphologies have been adopted as support materials for the Fe_2_O_3_ catalyst. For example, the utilization of ZnO nanotubes as a support material for P–Fe_2_O_3_ is known to boost the catalytic activity, as the 1-dimensional tube structure of the support oxides (ZnO) provides effective pathways for the charges. On the other hand, the doping of Fe_2_O_3_ by anions such as P has been found to increase the donor density and improve the charge transfer process. A ZnO-coated P-Fe_2_O_3_ nanostructure was fabricated using chemical bath deposition (ZnO nanotube), followed by solution processing (Fe) and chemical vapor deposition (for P doping). During the water splitting experiments, ZnO-coated P-Fe_2_O_3_ generated 10 mA cm^−2^ of current density at low OER overpotential at 250 mV, whereas the HER overpotential was observed at 139 mV. The applicability of ZnO/P-Fe_2_O_3_ was evaluated by testing the overall water splitting in two electrode systems, in which the HER and OER processes were effectively performed at a lower bias potential of 1.62 V. Furthermore, the prolonged electrolysis ability of the electrode over six days was established, indicating the stability and versatility of the ZnO-coated P-Fe_2_O_3_ bifunctional catalyst. Similarly, P-doped Fe_2_O_3_ linked to CoP was also explored for overall water splitting, including seawater electrolysis (discussed in later sections). Liu et al. [43] demonstrated the doping of both anion and metal ions into Fe_2_O_3_ for enhanced water-splitting applications. Zn was used as a cation, whereas S was employed as an anion dopant, which was simultaneously introduced into Fe_2_O_3_ linked to Fe_3_O_4_ on the iron foam. The Zn and S doped-Fe_2_O_3_/Fe_3_O_4_ (on iron foam) was found to undergo directional reconstruction into FeOOH to transform into Zn and S doped-Fe_3_O_4_-FeOOH (on iron foam). Even though Zn/S/Fe_3_O_4_-FeOOH showed the highest OER efficiency, its Fe_2_O_3_ counterpart showed significant water-splitting efficiency. Conclusively, doping Fe_2_O_3_ by anions and/or metal ions seems to be the key to enhancing the water-splitting activity and stability.

### 4.5. Fe_2_O_3_ in Heterojunction Systems

Constructing a heterojunction system with Fe_2_O_3_ offers significant advantages, e.g., providing support to the catalyst, acting as the main catalyst to support active sites, providing oxygen-deficient defects, etc., thereby improving the electrocatalytic activity. Specifically, the utilization of Fe_2_O_3_ as a support material for the catalyst is expected to decrease the quantity of loading of the main catalyst, which becomes vital in the case of expensive and rare metal-based catalysts. For example, the loading of a Co_3_O_4_ catalyst could be significantly reduced by using Fe_2_O_3_ as a support [44]. Even though experimental results have shown that Fe_3_O_4_ acts as a superior support to Co_3_O_4_ compared to Fe_2_O_3_, it still behaves more efficiently than the base Fe_2_O_3_ and Co_3_O_4_ electrodes, indicating the synergistic effect of using Fe_2_O_3_ as a support [44]. In the case of C-doped CoFe_2_O_4_ linked to Fe_2_O_3_, a significantly lower overpotential requirement was noted [45], that is, the C-doped CoFe_2_O_4_/Fe_2_O_3_ required 260 mV of overpotential to produce an OER current density of 100 mA cm^−2^ and an HER overpotential of 236 mV, indicating excellent catalytic activity for overall water splitting. Similarly, the conjugation of Mo in addition to Co (as an alloy) with Fe_2_O_3_ has shown promising water-splitting results [46] owing to its bifunctional catalytic properties. The fabrication of a CoMo-Fe_2_O_3_ heterojunction on a nickel foam involved two sections, in which Fe_2_O_3_ was first deposited on the nickel foam using the hydrothermal method, followed by CoMo alloy deposition using the electrodeposition technique. The Fe_2_O_3_ nanostructure on the nickel foam appeared to have a nanosheet morphology, whereas the CoMo alloy showed spherical microparticles distributed evenly on the Fe_2_O_3_ nanosheets. The unique two-dimensional structure with spherical particles in the CoMo-Fe_2_O_3_ system could result in increased penetration of electrolyte ions, leading to an increase in the number of reaction sites, and thus, enhanced electrocatalytic activity. The electrochemical characterization results of CoMo-Fe_2_O_3_ revealed an impressive current density value of 50 mA cm^−2^ for the OER process at the lowest overpotential of 266 mV. On the other hand, the 10 mA cm^−2^ of HER current was recorded at an potential value of 71 mV. The Tafel slope for OER and HER activity was found to be 54 and 85 mV dec^−1^, respectively. The practicality of the CoMo/Fe_2_O_3_ catalyst was demonstrated by using two electrode setups for alkaline water splitting that exhibited a current density of 10 mA cm^−2^ at a lower bias voltage of 1.5 V. A theoretical study on CoMo/Fe_2_O_3_ revealed the formation of an effective interface that facilitated efficient charge redistribution and transfer, leading to enhanced catalytic activity. Importantly, a remarkable stability of over 100 h was recorded under prolonged electrolysis conditions as a bifunctional catalyst. Alothman et al. [47] have proposed the Fe_2_O_3_@CuO heterojunction as a bifunction catalyst for water electrolysis. Using a simple hydrothermal method followed by calcination, Fe_2_O_3_@CuO catalysts were synthesized; these were then coated onto a nickel foam by forming an ink with nafion. The optimized Fe_2_O_3_@CuO electrode exhibited OER and HER overpotentials of 230 and 130 mV, respectively, reaching 10 mA cm^−2^ of current density. Furthermore, a Tafel slope of 54 and 77 mV dec^−1^ was observed in the OER and HER voltammograms, respectively.

Noble metal catalysts could be anchored to Fe_2_O_3_ to enhance its activity and bifunctionality and reduce costs. For example, Fe_2_O_3_ and IrO_2_ are well known OER catalysts; in contrast, their HER activity is rarely exploited. However, a composite of Fe_2_O_3_ and IrO_2_ was found to perform effectively as a HER catalyst, as demonstrated by Yang et al. [48]. A thermal decomposition method was used that effectively optimized the IrO_2_ and Fe_2_O_3_ content. The electrochemical measurements conducted in H_2_SO_4_ (0.5 M) indicated that the adsorption of hydrogen was more efficient on the IrO_2_/Fe_2_O_3_ composite compared to its individual counterparts, resulting in a shift of overpotential onset toward the 0 V vs. RHE, due to the Volmer–Heyrovsky mechanism. Similarly, Mosallaei et al. [49] developed a RuO_2_–Fe_2_O_3_ catalyst using the Ru-ionic complex. The proposed strategy differentiates itself from the reported methods by not using common metal salts for the oxide catalyst synthesis. In this approach, the synthesized Ru and Fe complex (Ru(then)_3_]_2_[Fe(CN)_6_] and [Ru(phen)_3_] [Fe(CN)_5_(NO)]) were calcined to yield the RuO_2_–Fe_2_O_3_ nanocrystal catalyst. A morphological analysis indicated good crystallinity of RuO_2_–Fe_2_O_3_ with an average particle size of 8–12 nm. In the OER and HER experiments, the RuO_2_–Fe_2_O_3_ catalyst exhibited an overpotential value of 292 and −148 mV, respectively, to produce a current density of 10 mA cm^−2^. The low Tafel slope values of 56.08 and −43 mV dec^−1^ were calculated from the OER and HER process LSV curves, respectively. The low charge transfer resistance at the electrolyte interface indicated the utilization of holes and electrons for OER and HER activity, confirming the superior bi-functional catalytic activity of the RuO_2_–Fe_2_O_3_ catalyst. A similar strategy was employed by Mosallaei et al. [50] to produce a RuO_2_–Fe_2_O_3_ catalyst using metal complexes. In their protocol, an impregnation method was used by utilizing [Ru(dmbpy)_3_]_3_[Fe(CN)_6_]_2_, an ionic pentanuclear complex, followed by thermal decomposition to yield RuO_2_–Fe_2_O_3_ on a reduced graphene oxide nanosheet support. The nanorod and cubic-shaped RuO_2_–Fe_2_O_3_ were found to be evenly distributed on the reduced graphene oxide nanosheets, indicating the presence of effective diffusion pathways via the superior conductivity and charge transfer properties of the reduced graphene oxide nanosheets during the water splitting reactions. Owing to their superior catalytic and nanostructure properties, the RuO_2_–Fe_2_O_3_ and highly reduced graphene oxide nanosheets deposited on glassy carbon electrode exhibited an OER and HER overpotential value of 386 and −239 mV and a Tafel slope value of 67 and −97 mV dec^−1^, respectively, in a 1 M KOH electrolyte. Furthermore, significant stability of the catalysts over 16 h was observed during the chronoamperometric measurements. Conclusively, the systematically arranged RuO_2_–Fe_2_O_3_ catalysts on highly reduced graphene oxide nanosheets were shown to offer significantly increased catalytic sites with increased reaction surface area and porosity, leading to a synergistic effect for enhanced overall water splitting.

Yan Sang, ref. [51] proposed a one-step hydrothermal fabrication method for a Fe_2_O_3_/NiO bifunctional catalyst for water splitting. The material morphology showed a systematic distribution of Fe_2_O_3_ nanoparticles (50–80 nm) on hexagonal-shaped NiO nanoflakes. The LSV measurements in 1.0 M KOH indicated that Fe_2_O_3_/NiO required an OER overpotential of 224 mV and a HER overpotential value of 187 mV to achieve 10 mA cm^−2^ of current density. Furthermore, a Tafel slope of 20.0 and 53.8 mV dec^−1^ was observed from the LSV data of the OER and HER activity, respectively. Importantly, Fe_2_O_3_/NiO demonstrated its bifunctional catalytic activity by exhibiting alkaline overall water splitting at a cell potential value of +1.63 V vs. SHE and over 20 h of stability. The enhanced OER and HER performance of the Fe_2_O_3_/NiO catalyst was ascribed to the optimized electronic structure of heterojunctions, owing to the optimum level of oxygen vacancies in Fe_2_O_3_ that favor efficient charge transfer to improve the catalytic activity. The introduction of WO_3_ into the Fe_2_O_3_/NiO catalyst was found to decrease the required overpotential [52]. However, unlike Fe_2_O_3_/NiO, the WO_3_/Fe_2_O_3_/NiO catalyst functioned as only an OER catalyst, rather than as a bifunctional catalyst. The adopted synthesis method involved simultaneous hydrothermal treatment, comprising an etching reaction and the decomposition of metal ions. Such a synthesis approach caused the regulation of the intrinsic electronic properties of the catalysts due to the presence of high-density heterometal-oxygen bridge sites at the interface. A remarkably low overpotential value of +211 mV was shown to achieve 100 mA cm^−2^ of OER current density. Furthermore, a low Tafel slope of 39.5 mV dec^−1^ indicated excellent catalytic properties, and a stability of over 100 h was observed. A detailed mechanistic study indicated that the presence of W in the catalysts sped up the oxidation of Ni to the 3+/4+ state from the 2+ state due to the partial electron transformation from the high valence W^6+^ state. Among the reaction intermediates, a weaker adsorption energy of OH* was expected on WO_3_ and a stronger one on Fe_2_O_3_. Being a stronger Lewis acid, W^6+^ readily oxidizes the neighboring Ni^2+^ and Fe^3+^ via the withdrawal of electrons. This mechanism optimizes the adsorption energies of the reaction intermediates and thus enhances the catalytic activity [52]. Conclusively, the presence of W has been revealed to be the key factor in achieving the lower overpotential of OER activity in Fe_2_O_3_/NiO catalysts. However, in the case of Fe_2_O_3_/Ni(OH)_2_ and Fe_2_O_3_/NiFe alloy nanoparticles, a slightly higher OER overpotential was observed [53,54] indicating that anchoring Fe_2_O_3_ with NiO and WO_3_ offers efficient OER catalysis.

The conjugation of Fe_2_O_3_ with MnO enhances the OER activity, as reported by Kim et al. [55]. A simple sol-gel technique was proposed to synthesize individual and heterojunctions of MnO and Fe_2_O_3_. In their electrochemical investigation, the materials were coated onto a Ni foam substrate (as a current collector) in the electrolyte. The electrochemical tests conducted in alkaline media indicated that the MnO–Fe_2_O_3_ heterojunction reached 10 mA cm^−2^ of current density at a bias potential of +1.60 V vs. SHE; this was ascribed to the overpotential value of +370 mV. Furthermore, a 66 mV dec^−1^ Tafel slope was recorded with a cycle stability of over 1000 cycles. Even though the overall overpotential requirement at 10 mA of current density was not high enough compared to the literature, the heterojunction was found to perform significantly better than its individual counterparts. Notably, the underlying mechanism indicated the synergistic effect of Mn and Fe, which induce the oxidation of O_2_ and the reduction of H_2_, respectively.

**Figure 6:**
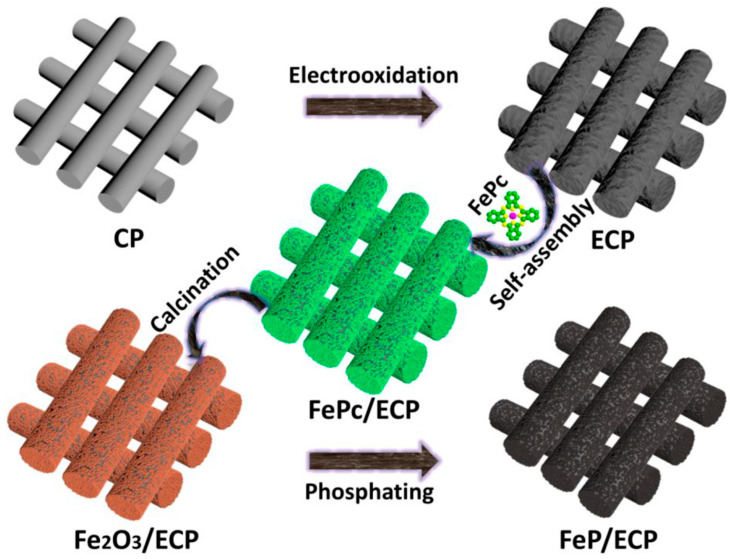
Synthesis protocol of Fe_2_O_3_ and conversion to FeP on an ECP (electro-oxidized carbon paper) substrate. Adapted with permission from [56]. Copyright [2018] American Chemical Society.

**Figure 7:**
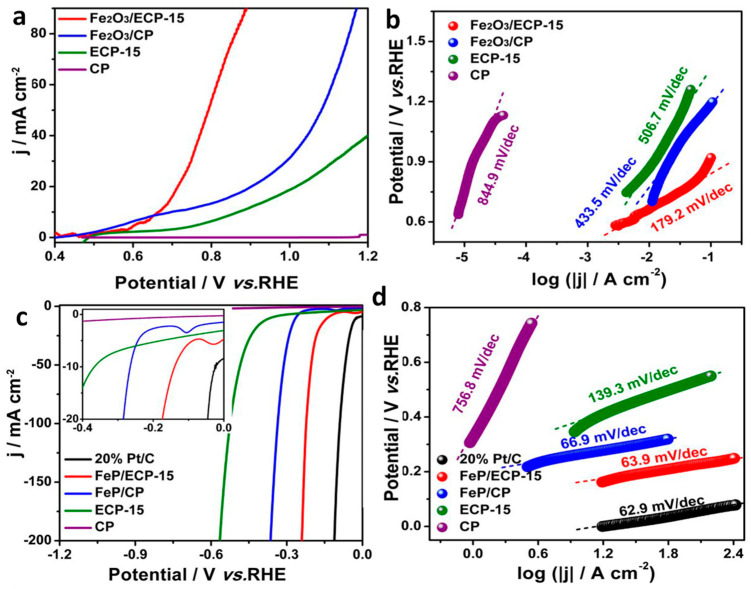
(**a**,**c**) LSV and (**b**,**d**) Tafel plots of Fe_2_O_3_ and FeP systems on CP and ECP substrates. Adapted with permission from [56]. Copyright [2018] American Chemical Society.

Recently, Fe_2_O_3_ has been used to oxidize hydrazine in an asymmetric electrochemical cell with a FeP cathode to produce H_2_ [56]. In that report, electrolyzed carbon paper was used as a substrate and current collector. The Fe_2_O_3_ nanostructures were deposited on the substrate (referred to as electro-oxidized carbon paper) via the self-assembly of iron phthalocyanine, followed by pyrolysis in air (Figure 6). For the cathode electrode, the Fe_2_O_3_ deposited on the substrate was further treated with NaH_2_PO_2_ in the CVD system for phosphidation, yielding FeP on electro-oxidized (EO) carbon paper (Figure 6). Both the Fe_2_O_3_ and FeP deposited on the EO carbon paper appeared to show a mosslike morphology, in which the latter showed a weaker surface due to the Kirkendall effect. The morphology appeared to consist of closely packed nanocrystals which provided effective catalytic sites, whereas the porous architecture led to the effective diffusion of electrolyte ions and provided effective gas evolution pathways. In the absence of hydrazine, the OER overpotential was observed at +1.56 V vs. SHE to produce 10 mA cm^−2^. Both Tafel plots and impedance values showed decreased slope and charge transfer resistance values, respectively indicating the advantages of coupling Fe_2_O_3_ and FeP in a two-electrode system for hydrazine oxidation for H_2_ production. In particular, to reach a hydrazine oxidation current density of 10 mA cm^−2^ at the Fe_2_O_3_ anode, a potential of 664 mV was required with a Tafel slope value of 179.2 mV dec^−1^ (Figure 7a,b). In contrast, a HER current density of 10 mA cm^−2^ at FeP was achieved with an overpotential of +77 mV with a Tafel slope value of 63.9 mV dec^−1^ (Figure 7c,d). The electrochemical tests conducted in two-electrode setups consisting of a Fe_2_O_3_ anode and a FeP cathode on electrooxidized (EO) carbon paper indicated an efficient hydrazine oxidation (at the anode) current density of 10 mA cm^−2^ at an impressively lower overpotential of +930 mV, along with the evolution of H_2_ gas at the FeP cathode. This study indicated that an alternate reaction for water oxidation with Fe_2_O_3_ could be beneficial in achieving higher current densities with lower overpotentials. Such systems are believed to represent a key strategy in the fabrication of membrane-less or decoupled electrolysis systems.

The Fe_2_O_3_ and FeP heterojunction systems could be synthesized using a hydrothermal method with iron and red phosphorus as precursors [57]. A study of the morphology of the hydrothermally synthesized Fe_2_O_3_-FeP revealed that FeP consists of a nanorod structure, whereas Fe_2_O_3_ forms as cubic nanoparticles. The morphology showed the presence of a porous system that could be utilized for the effective diffusion of electrolyte ions and the removal of the produced gases. The electrochemical experiments conducted in an alkaline electrolyte (1 M KOH) indicated an impressive OER overpotential of +264 mV to achieve 10 mA cm^−2^ of current density. Furthermore, the low value of the Tafel slope and resistance value (at the electrode/electrolyte interface) indicated the excellent catalytic activity offered by the Fe_2_O_3_/FeP heterojunction. A prolonged water splitting test indicated a stability of over 12 h with two electrode setups, with a potential requirement of only +1.65 V vs. SHE.

Unlike FeP anchored to Fe_2_O_3_ systems, FeS linked to Fe_2_O_3_ showed a decreased efficiency, indicating that FeP serves as an effective junction material compared to FeS in terms of OER activity [58]. In contrast, FeS anchored to Fe_2_O_3_ demonstrated a significantly lower overpotential in the study reported by Guo et al. [59]. In that study [59], the FeS/Fe_2_O_3_ catalyst (on the iron foam) was fabricated using a semi-sacrificial template approach that involved a solvothermal approach to produce Fe_2_O_3_, followed by exposure to air and treatment with a sulfide precursor to form FeS. The FeS/Fe_2_O_3_ showed a nanosheet morphology with an average size of 0.8 µm. A high-resolution TEM analysis confirmed nanojunction formation between the Fe_2_O_3_ and FeS, with good crystallinity and uniform distribution of elements. The formation of heterojunctions between Fe_2_O_3_ and FeS offered an effective interface with a tuned electronic structure, leading to effective an electron-proton transfer process that increased the number of active sites with improved stability and OER activity. The optimized FeS/Fe_2_O_3_ system generated 10 mA cm^−2^ of current density at a lower overpotential value of +266.5 mV. Moreover, enhanced stability of over 50 h was recorded with a Tafel value of 51.17 mV dec^−1^. The efficient catalytic activity and stability indicated the potential applicability of this material in industrial scale hydrogen production via electrolysis. Similar to iron sulfide, iron selenide anchored to Fe_2_O_3_ is known to exhibit excellent electrocatalytic water-splitting activity, as demonstrated by Sohail et al. [60]. As such, Fe_2_S_3_ has been used in OER. However, unlike FeS-Fe_2_O_3_, Fe_2_Se_3_/Fe_2_O_3_ shows a remarkably low overpotential value of +160 mV for OER activity at 20 mA cm^2^, implying the lowest applied potential of +1.31 V vs. SHE. Indeed, the overpotential value exhibited by Fe_2_Se_3_/Fe_2_O_3_ was found to be the lowest for the Fe_2_O_3_-based electrode. Furthermore, excellent stability over 24 h was observed during chronoamperometric tests, with a stable current density of 65 mA cm^−2^ at a bias potential of +1.65 V vs. SHE.

### 4.6. Fe_2_O_3_ Supported on Carbon Materials

Fe_2_O_3_ is known for its efficient conductivity compared to other metal oxides. Hence, providing a support material becomes essential for the diffusion/transfer of charges during catalysis. In such cases, the tuning of the catalyst morphology and the conductive nature of the support materials are vital for improved electrocatalytic activity. In the case of Fe_2_O_3_, limiting its nanomorphology by strategically tuning the crystal size and nanoarchitecture is effective due to its limited carrier path length. Specifically, limiting the nanostructure of Fe_2_O_3_ to one or two dimensions can boost the catalytic activity by orders of magnitude compared to its microstructures [82]. In addition, providing a highly conductive platform such as graphene (including other carbon supports) to Fe_2_O_3_ will further support the catalytic activity and stability by effectively and rapidly diffusing the charges that are responsible for catalytic reactions and conductivity [83,84,85]. For example, anchoring Fe_2_O_3_ hollow nanorods to carbon nanotubes (CNT) has been found to enhance the OER activity [61]. A Fe_2_O_3_-CNT junction could be fabricated via the co-precipitation method using urea. Even though the achieved overpotential, Tafel slope, and stability values were +383 mV, 68 mV dec^−1^, and 12 h, respectively, the values were less significant compared to those in other reports. The strategy of using a carbon support may be a solution to overcome two main challenges, i.e., the limited conductivity of Fe_2_O_3_ and the poor penetration of the electrolyte on the surface of the catalyst. Furthermore, the introduction of CNT supports the underlying faradaic process by decreasing the resistivity. As a result, the Fe_2_O_3_-CNT performed better as a catalyst than pristine Fe_2_O_3_.

Zang et al. [62] investigated the OER activity of Fe_2_O_3_ anchored to Fe-N-doped carbon nanosheets. A high-temperature facile pyrolysis (1000 °C) approach was proposed to yield mesoporous Fe/Fe_2_O_3_-Fe-N-doped carbon nanosheets using shrimp shells. The optimized catalyst on GCE exhibited an OER current density of 10 mA cm^−2^ at +0.69 V (vs. Ag/AgCl) and a Tafel slope of 77.5 mV dec^−1^ with a stability of 10 h. Notably, the OER performance of the Fe/Fe_2_O_3_-Fe-N-doped carbon nanosheets was significant compared to commercial RuO_2_. Similarly, Fe_2_O_3_ deposited on graphitized-carbon nitride nanocomposites was used in OER with alkaline media [63]. The electrochemical results were less significant compared to those of Fe/Fe_2_O_3_-Fe-N-doped carbon nanosheets. However, Fe_2_O_3_ anchored to N-doped carbon nanomaterial showed HER activity in alkaline media [64]. That is, unlike other carbon supports on Fe_2_O_3_, the catalyst, as reported by Jiang et al. [64], showed HER activity. An electrochemical analysis performed in 1 M KOH revealed a HER current density of 10 mA cm^−2^ at an overpotential of +245 mV with a Tafel slope value of 76.6 mV dec^−1^. Furthermore, excellent stability of the catalyst was observed up to 48 h of water splitting.

The introduction of Ni_3_S_2_ along with Fe_2_O_3_ anchored to N-doped carbon composites has been shown to yield excellent OER and HER activities [65]. Even though Ni_3_S_2_ is well known for its catalytic activity, its lifespan tends to be short due to ineffective coupling to the back contact or substrates. To overcome this challenge, the utilization of Fe_2_O_3_ and N-doped carbon composite has been proposed. A facile thermal decomposition approach was used with essential precursors to fabricate a Ni_3_S_2_/Fe_2_O_3_/N-doped carbon composite. The characterization of the catalyst-deposited nickel foam revealed nanosheets of Ni_3_S_2_ containing Fe_2_O_3_ and N-doped carbon particles. An electrochemical analysis conducted in 1 M KOH indicated that the Ni_3_S_2_/Fe_2_O_3_/N-doped carbon composite exhibited an impressive OER overpotential of 188 mV for 52 mA cm^−2^ of current density, along with a Tafel slope value of 64.3 mV dec-1. In addition, about 10 mA cm^−2^ of HER current density was produced at an overpotential value of +74 mV with a Tafel slope of 115.8 mV dec^−1^. Importantly, the electrode composite in the bifunctional mode in two electrode systems was found to achieve about 100 mA cm^−2^ of current density at a bias potential of +1.605 V vs. SHE, which was supplied using an external battery. The superior catalytic activity achieved in this report indicates that the utilized synthesis approach and the composite anchored to Ni_3_S_2_ result in a strong coupling of the catalyst to the nickel foam substrate.

The simultaneous deposition of Fe_2_O_3_ and graphene oxide on a nickel foam substrate can be achieved using the co-deposition method [66]. The approach involves separate graphene oxide and Fe_2_O_3_ (sugar-cubic) synthesis using a modified Hummers method and hydrothermal method, respectively, followed by electrodeposition to deposit the materials on the Ni foam. An electrocatalytic characterization conducted in 1 M KOH showed an overpotential value of +313 mV to generate an OER current density of 100 mA cm^−2^; this was ascribed to the Tafel slope of 81 mV dec^−1^. The improved OER overpotential value of Fe_2_O_3_/graphene oxide was ascribed to the increased surface area of the nitrogen-doped graphene with effective mechanical properties that provide stability and enhanced active sites during electrolysis. Furthermore, the presence of uniformly distributed Fe_2_O_3_ nanocrystals on nitrogen-doped graphene offered increased reaction sites, favoring an effective interface at the electrolyte and providing a solid foundation for charge collection and diffusion/transfer. Likewise, dispersing Fe_2_O_3_ on MWCNT could also provide similar advantages to graphene in terms of increasing the OER activity of Fe_2_O_3_ [67]. In other words, the presence of MWCNT as a support for Fe_2_O_3_ favors the adsorption of OER intermediates that drive efficient charge transfer kinetics. Surface-functionalized multiwall carbon nanotubes act as an effective support to the uniform deposition of Fe_2_O_3_, which was achieved by pulsed laser ablation deposition. The LSV measurements were noted to produce 10 mA cm^−2^ of current density at a lower overpotential of +310 mV and a Tafel slope of 20.35 mV dec^−1^, confirming the superior OER activity of Fe_2_O_3_/MWCNT compared to its counterparts. The presence of MWCNT decreased the energy barriers of the OER intermediates, which are rate-determining. However, a limited stability of only 10 h was observed, which may not be significant compared to other electrode combinations that support Fe_2_O_3_. Therefore, among the tested carbon supports for Fe_2_O_3_, Ni_3_S_2_ anchored to N-doped carbon composites was found to yield superior OER activity, in addition to HER activity.

### 4.7. Fe_2_O_3_ Linked to MOFs

**Figure 8:**
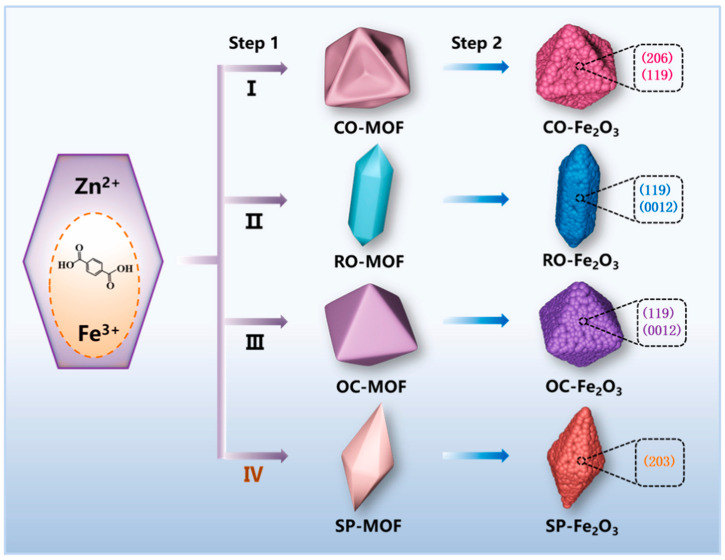
Synthesis of morphologically tuned Fe_2_O_3_ from MOF. CO—a concave octahedral structure, RO—a nanorod-like structure, OC—octahedron, SP—a spindle structure. Reused with permission from [68]. Copyright 2021, with permission from Elsevier.

Recently, MOFs have gained significant attention in energy applications owing to their unique architecture, i.e., containing organic ligands linked to metal clusters [86]. MOF offers tunable porosity with an enhanced specific surface area that could be engineered into specific morphologies depending on the application needs. Using the unique properties of Fe_2_O_3_, Niu et al. [68] proposed FeOx node-based MOFs with engineered intrinsic active sites and architectures with enhanced reaction areas that exhibited excellent OER, HER, and ORR activities. The procedure involves the simple solvothermal method that allows extensive tuning of bonding between the Fe and 1,4-dicarboxybenzene ligands to be undertaken via the utilization of different solvent systems and a wide range of metal sources. Such tuning provides effective control over the selective or desired morphologies, such as concave octahedral, rod-like, spindle-like, and octahedron-like, offering desirable properties (Figure 8).

**Figure 9:**
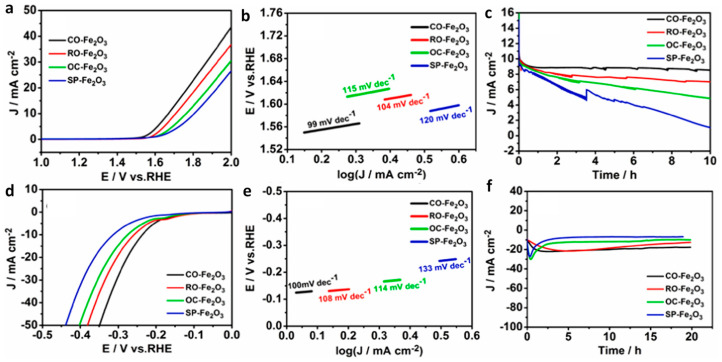
(**a**,**d**) LSV curves (**b**,**e**) Tafel plots and, (**c**,**f**) I-t plots for morphologically tuned Fe_2_O_3_ in terms of OER and HER activity. Reused with permission from [68]. Copyright 2021, with permission from Elsevier.

Furthermore, a pyrolysis step was proposed to yield a graphite nanocomposite of Fe_2_O_3_ that displayed a wide range of exposed crystal planes having unique properties and reaction capabilities with retained original morphologies (Figure 8). Figure 9 shows the LSV, Tafel slope, and I-t stability plots of concave octahedral, rod-like, spindle-like, and octahedron-like MOF-Fe_2_O_3_ for OER and HER applications. Among the explored structures, the concave octahedral form of Fe_2_O_3_—MOF was found to possess an enhanced active site, owing to the favorable facets for improved electrocatalytic OER and HER activity, with enhanced stabilities, as shown in Figure 9 [68]. Importantly, the results of this report indicate the applicability of Fe_2_O_3_ in MOF systems in terms of electrocatalytic activity.

### 4.8. Sea/Domestic Wastewater Electrolysis

**Figure 10:**
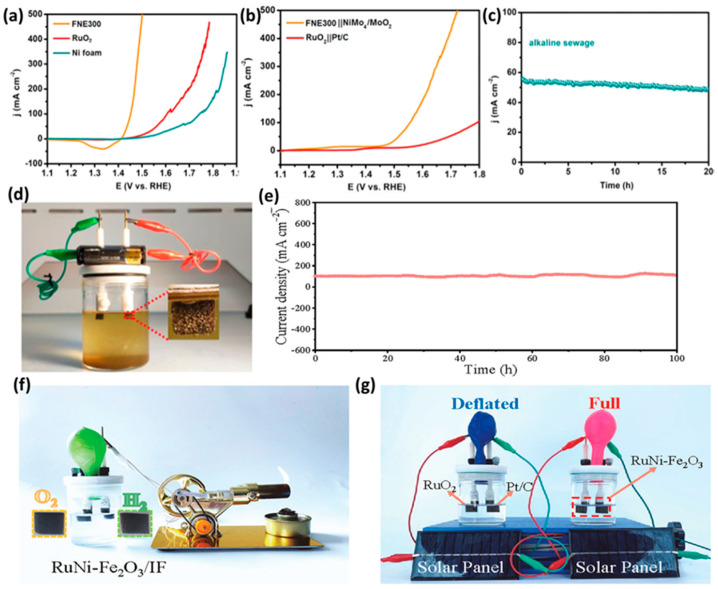
(**a**) LSV curves of the FNE300 (FNE300 represents Fe_2_O_3_/NiO heterojunction system) compared to the standard RuO_2_ and Ni foam substrate, measured using an alkaline sewage electrolyte. (**b**) LSV curves standard RuO_2_||Pt/C and FNE300||NiMo_4_/MoO_2_ in standard electrolyzers system measured using an alkaline sewage electrolyte. The (**c**) I-t plot (**d**) photographic image of FNE300||NiMo_4_/MoO_2_ system in electrolysis condition. Adapted with permission from [69]. Copyright [2021] American Chemical Society. (**e**) I-t plot of RuNi-Fe_2_O_3_ measured in alkaline seawater showing incredible stability. (**f**) The two-electrode setup for seawater electrolysis. (**g**) A comparison of the electrolysis activity of RuNi-Fe_2_O_3_ with a standard RuO_2_||Pt/C system powered using commercial solar panels. Reused with permission from [71]. Copyright [2022], with permission from Elsevier.

Sources of freshwater around the globe are valuable and limited, and thus, the utilization of seawater and domestic sewage in electrolysis is encouraged on the basis of cost efficiency and resource availability. However, the electrochemical water splitting of seawater is challenging due to the presence of unwanted and interfering ions, decreased conductivity, poisoning, and corrosion of the electrodes/catalysts [87,88]. The presence of chloride anions leads to the evolution of chlorine in the OER process that causes corrosion, poisons the anodic surface through the formation of insoluble precipitates, and compromises efficiency [89]. To inhibit the evolution of chlorine and selectively perform OER, a systematically designed catalyst needs to be implemented. Li et al. [69] proposed seawater and domestic sewage water electrolysis using a Fe_2_O_3_/NiO interface. A simple chemical bath deposition method was proposed for the synthesis of Fe_2_O_3_ on Ni foam-producing porous structures. The characterization of the catalyst indicated the formation of a FeNi oxide interface that exhibited superior OER activity. In a 0.1 M KOH electrolyte, the Fe_2_O_3_/NiO exhibited a 10 mA cm^−2^ current density at an overpotential value of +182 mV but also a high current density of 1000 mA cm^−2^ at an overpotential of +267 mV. Both the Tafel slopes and EIS measurements indicated low slope and charge transfer resistance values, respectively. An impressive stability of over 50 h was recorded in both seawater and domestic sewage water, indicating the versatility of the Fe_2_O_3_/NiO catalyst. The superiority of Fe_2_O_3_/NiO was further tested in two electrode setups using seawater and alkaline domestic sewage. For this, a NiMo_4_/MoO_2_ electrode was used as a cathode and compared with Pt/C in terms of practicality. With alkaline electrolytes including NaCl and sea water-containing mixtures, the Fe_2_O_3_/NiO catalyst performed significantly better compared to commercial RuO_2_ OER and Pt/C HER electrode systems, indicating exceptional selectivity of Fe_2_O_3_/NiO in the OER process. Furthermore, a stability of over 50 h was observed, indicating no apparent corrosion of the electrodes in Cl^−^ containing electrolytes. Similarly, the potential application of Fe_2_O_3_/NiO was tested in alkaline sewage electrolysis, as demonstrated in Figure 10. Interestingly, to perform OER with 100 mA cm^−2^ in alkaline sewage system, the Fe_2_O_3_/NiO utilized an overpotential of +222 mV, which was observed to be lower than that of the standard RuO_2_ OER catalyst, both in three- and two-electrode setups (Figure 10a,b). With an elevated current density of 500 mA cm^−2^, the bias potential was noted to be +1.752 V vs. SHE, which confirmed the pilot scale industrial feasibility of the Fe_2_O_3_/NiO catalyst. Mainly the catalyst performance was found to be consistent, despite the high chemical oxygen demand; this confirmed the anti-poisoning characteristic of Fe_2_O_3_/NiO as a catalyst. A photographic image of a Fe_2_O_3_/NiO anode catalyst connected to standard battery with a NiMo_4_/MoO_2_ cathode catalyst is shown in Figure 10, confirming the formation of gas bubbles. The I-t plot shown in Figure 10d further confirms the stability of Fe_2_O_3_/NiO for more than 30 h of operation, indicating the catalytic performance and selectivity of the catalyst.

Cui et al. [71] proposed an effective and cost-effective method for the synthesis of a Ru, Ni-doped Fe_2_O_3_ bifunctional catalyst for alkaline and seawater splitting. The synthesis protocol involves the use of precursors in autoclave and hydrothermal synthesis with systematic optimization regarding the ratio of Ni and Ru. The optimized Fe_2_O_3_ bifunctional catalyst exhibited 100 mA of OER and HER current density at an overpotential value of +329 and +75 mV, respectively, in a 1.0 M KOH electrolyte. In a two-electrode setup as the bifunctional electrode mode, the RuNi-Fe_2_O_3_ required a very low applied potential of +1.66 V vs. SHE in the standard alkaline electrolyte. Importantly, in the seawater electrolyte, a significantly low overpotential of +1.73 V vs. SHE was optimized, indicating the effectiveness of the catalyst in practical applications. The selectivity and stability were further proved by testing the RuNi-Fe_2_O_3_ for long-term seawater electrolysis, as shown in Figure 10e. The I-t plot (Figure 10e) suggests excellent stability over 100 h of seawater splitting, indicating the superior performance of RuNi-Fe_2_O_3_ as a bifunctional catalyst. The standalone device shown in Figure 10f shows a two-electrode setup with a RuNi-Fe_2_O_3_ bifunctional catalyst for seawater splitting. Furthermore, using commercial solar panels, the electrode system was utilized to perform seawater electrolysis using a RuNi-Fe_2_O_3_ bifunctional catalyst. The efficacy was compared with that of a standard RuO_2_-Pt/C electrode system, as shown in Figure 10f. The rapid evolution of gas bubbles, along with the increase in balloon volume, confirmed that the proposed catalytic system could be effectively employed in seawater electrolysis and could replace the PGM electrodes in alkaline water electrolysis systems.

P-doped Fe_2_O_3_ decorated on CoP could be utilized to split seawater into H_2_ and O_2_, as demonstrated by Cui et al. [70]. Specifically, P-Fe_2_O_3_ was utilized to overcome the poor H–OH moiety dissociation on CoP, as well asl the inefficient desorption of intermediate molecules. Nanosheets of P-Fe_2_O_3_-CoP were fabricated using two two-step processes involving hydrothermal and gas-phase phosphorization procedures. During the water splitting process, the presence of P as a dopant was found to fulfill two main purposes, i.e., the introduction of an essential interfacial structure and defects. The optimized catalyst exhibited superior catalytic activity compared to the standard RuO_2_ catalyst. In seawater and freshwater electrolytes (containing 1 M KOH), a significantly low overpotential of +270 and +250 mV to generate 10 mA cm^−2^ of current density was observed. Furthermore, when employed as a bifunctional catalyst in a two-electrode electrolysis assembly, the P-Fe_2_O_3_-CoP catalyst exhibited a required bias potential of +1.61 vs. SHE for fresh water and about +1.65 V vs. SHE for seawater splitting reactions (for 10 mA cm^−2^). The low Tafel slope values, decreased electrode/electrolyte charge transfer resistance, and prolonged stability further confirmed the efficacy of the P-Fe_2_O_3_-CoP catalyst in fresh water and seawater electrolysis.

### 4.9. Influence of Magnetic Fields on Catalysts

The utilization of magnetic fields during electrolysis is known to provide a significant catalysis effect by accelerating the OER and HER activities [90,91,92]. The application of magnetic fields during electrolysis could exert different effects on electrolysis systems, improving the efficiency by providing the electrocatalytic effect, magnetohydrodynamic influence, magnetothermal effect, bubble management, magnetic moment alignment, spin selective effect [93,94], etc. Specifically, the formation of gas bubbles at the electrode surface during electrolysis causes decreased efficiency. In the electrolysis industry, an estimated energy loss of ~40% is expected due to the accumulation and inefficient removal of bubbles from the electrode surface [95]. The ineffective removal of gas bubbles may block the electrode surface, causing increased ohmic resistance and inhibiting facile mass transfer and nucleation [96].

**Figure 11:**
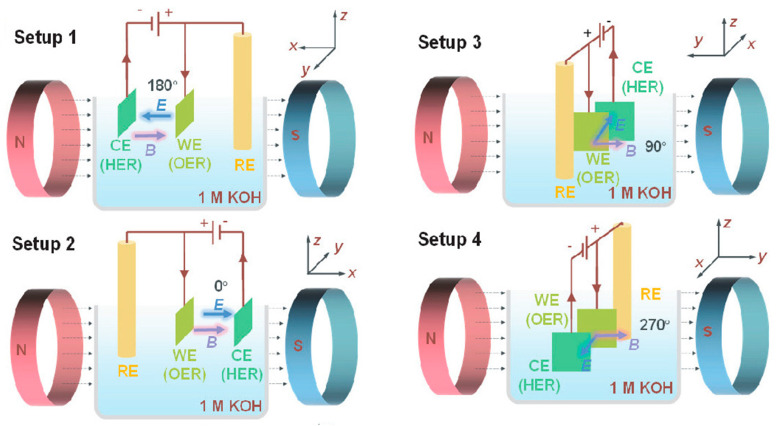
The different experimental setups tested for the application of magnetic fields to an electrode system. Reused with permission from [97]. Copyright 2023, with permission from Elsevier.

**Figure 12:**
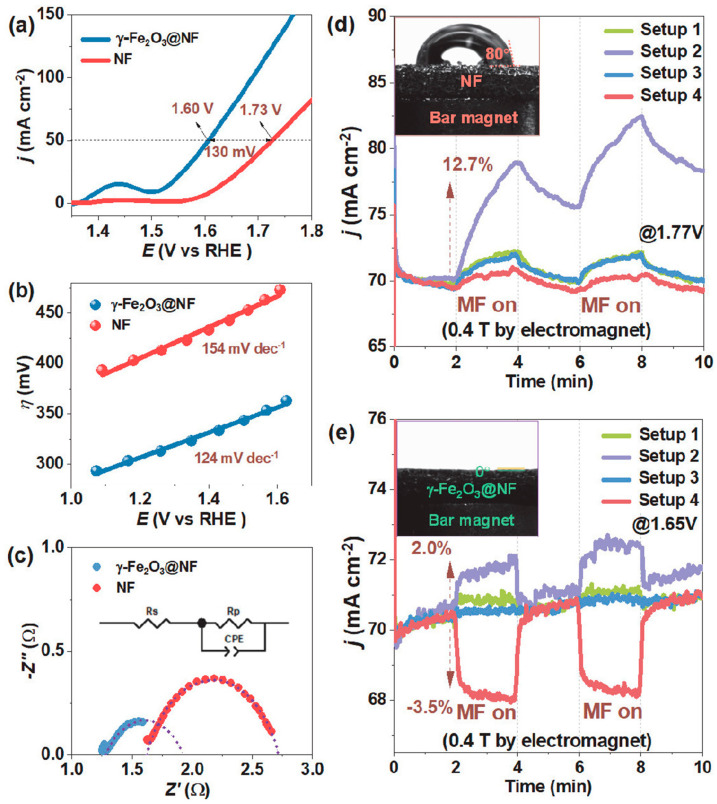
(**a**) LSV, (**b**) Tafel plot, and (**c**) Nyquist plots of γ-Fe_2_O_3_ (measured at a bias voltage of 1.6 V) on nickel foam for OER, measured in 1 M KOH at a scan rate of 5 mV s^−1^. Chronoamperometric curves of γ-Fe_2_O_3_ coated nickel foam measured at a bias potential of (**d**) 1.77 and (**e**) 1.65 V, and inset photographic image showing the respective contact angles under the applied 0.4 T magnetic field. Reused with permission from [97]. Copyright 2023, with permission from Elsevier.

Recently, Li et al. [97] demonstrated an effective route of harnessing magnetic fields in catalytic materials to enhance the OER process. This was successfully achieved by employing γ-Fe_2_O_3_ electrodes, owing to their magnetic properties. Specifically, cubic spinel structured γ-Fe_2_O_3_ (maghemite) is widely known for its strong magnetic properties and is widely employed as a permanent magnetic material [98]. The authors proposed a super-hydrophilic γ-Fe_2_O_3_ hydrosol with tuned surface wettability and magnetism for application under magnetic fields. This was achieved by coating the γ-Fe_2_O_3_ hydrosol on different substrates having unique magnetic and surface properties, such as carbon cloth (CCloth), nickel foam (NF), heat-treated carbon cloth (CCloth-Treated), and carbon paper, using a simple dip-coating method. Figure 11 shows the different setups used to induce magnetic fields in Fe_2_O_3_ electrodes. For instance, the catalytic activity of super-hydrophilic γ-Fe_2_O_3_ on NF was confirmed by LSV, Tafel plot, and EIS measurements, as shown in Figure 12a–c, respectively. The γ-Fe_2_O_3_ exhibited a cathodic shift in the onset potential in the LSV and required only +1.6 V vs. SHE to achieve 50 mA cm^−2^ of current density, whereas only Ni foam required a significant higher potential of +1.73 V vs. SHE to achieve the same result. The cathodic shift in potential confirmed that the presence of super-hydrophilic γ-Fe_2_O_3_ favored OER activity over bare Ni foam. Furthermore, the super-hydrophilic γ-Fe_2_O_3_ showed a decreased Tafel slope (Figure 12b) and polarization resistance (Figure 12c) compared to the untreated Ni foam. In addition, an 80° contact angle was observed for bare Ni foam (inset of Figure 12d), whereas the γ-Fe_2_O_3_ showed a super-hydrophilic characteristic (inset of Figure 12e). Conclusively, the surface super-hydrophilic property of γ-Fe_2_O_3_ ensures the effective release of O_2_ bubbles at the surface, along with the efficient and rapid transport of the electrolyte toward the electrode surface, leading to improved OER activity.

The chronoamperometric measurements performed (γ-Fe_2_O_3_/Ni-foam) in the presence of a magnetic field showed impressive results, indicating the importance and advantages of the use of magnetic fields. Four different setups were tested based on the magnet field input direction and system configuration, as shown in Figure 12. The experimental results confirmed that irrespective of the setup, a significant increase in current density was observed under the influence of a magnetic field. Among the setups, the second system showed a comparatively more significant influence of magnetic fields on OER activity. In the case of diamagnetic substrates, negative results were obtained. Importantly, the use of magnetic fields in electrocatalytic systems was found to provide an added Lorentz and Kelvin force for the effective removal of O_2_ bubbles with efficient transport of electrolytes based on the implemented setup and substrates [97].

### 4.10. Fe_2_O_3_ in AEMWE Systems

**Figure 13:**
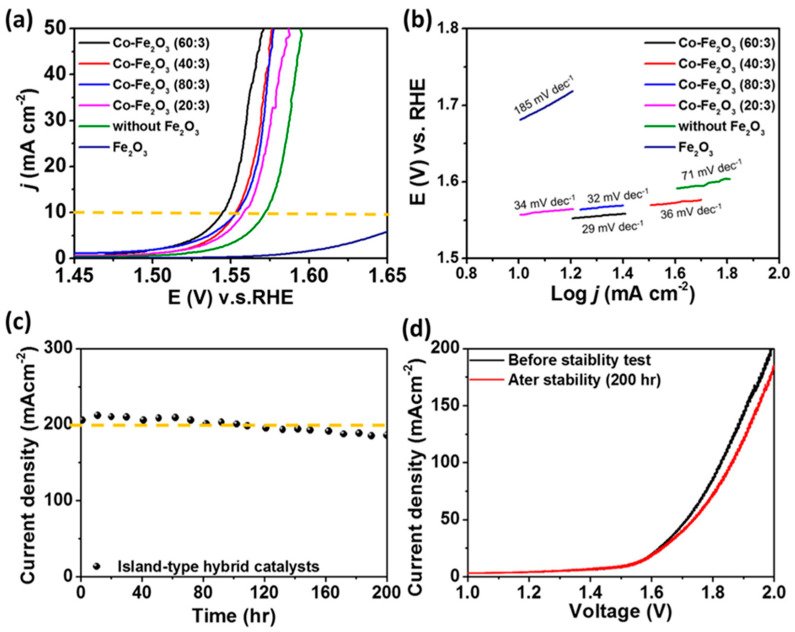
(**a**) Voltammograms and (**b**) Tafel slopes of Co-Fe_2_O_3_ catalyst with different ratios. (**c**) The stability I-t plot of Co-Fe_2_O_3_ of 60:30 ratio with (**d**) LSV plot measured before and after the stability tests. Reproduced with permission from [91].

AEMWE has emerged as one of the promising technologies that balances the advantages of both AWE and PEMWE into a single system. AEMWEs are operated in moderately weak alkaline conditions compared to AWE and integrated into membrane-based electrolyzer configurations like PEMWE. Unlike PEMWE, AEMWE may be used in non-PGM-based catalysts without any requirement for acidic electrolytes. Hence, the catalysts integrated into AEMWE are crucial for efficiency and stability. In AEMWE, the cracking of catalysts was found to be one of the limitations that hinders long-term activity and stability. Recently, Chen et al. [72] indicated the importance of Fe_2_O_3_ (with Co) as an island-type hybrid catalyst in AEMWE electrolysis that significantly inhibits the cracking of the catalysts. Using a systematically developed microwave-assisted hydrothermal technique, an island-type hybrid catalyst containing a controlled ratio of Fe_2_O_3_ and Co was synthesized. In the AEMWE system, the optimized Fe_2_O_3_/Co was used as an anode catalyst, whereas Pt/C was used as a cathode catalyst, which was loaded onto a carbon cloth support using a hand painting technique. The commercially available Fumapem FAA-3 membrane (Fumatech, Bietigheim-Bissingen, Germany) was used in the membrane electrode assembly (MEA) stack. The characterization results showed that the Fe_2_O_3_ acted as a support on the Co catalyst, providing a nanoscale gap for the effective removal of product gases with the electrolyte fill-ups. The efficiency of the catalyst was found to be dependent on the ratio of Fe_2_O_3_ and Co, as shown in the LSV and Tafel plots (Figure 13a,b). Electrolysis testing carried out in 1 M KOH (Co-Fe_2_O_3_ of 60:30 ratio) produced an impressive current density of 200 mA cm^−2^, which was found to be much higher than the catalyst without the Fe_2_O_3_ support layer. Stability studies conducted over a longer electrolysis period indicated an impressive stability over 200 h (Figure 13c), with a retention of over 90% of the initial current density. Moreover, the LSV plots measured before and after the stability tests indicated no significant difference, indicating the superior stability of the Co-Fe_2_O_3_ catalyst (Figure 13d).

### 4.11. Fe_2_O_3_ Supported Ceramics in SOEC

The solid-state structure of SOEC systems offers significant advantages over other hydrogen electrolyzer technologies, e.g., a unique ion diffusion mechanism, high electrode reactivity hydrogen purity, simplistic design, and possible commercial applicability [92]. The solid ceramic electrolyte in SOEC systems that separates the anode from the cathode compartments and provides effective diffusion pathways to O^2−^ ions is one of the important components that determines the overall efficiency and device stability. A wide range of ceramic electrolytes have been proposed in the last few years. Among the electrolytes, yttria-stabilized zirconia (YSZ) has been widely employed, owing to its low conductivity for electrons. In SOEC systems, the oxygen/cathode electrode materials play an important role and are generally doped with Sr for efficient oxygen ion transport and overall cell performance. The use of strontium-doped materials often aligns well with the other components of the SOEC, such as YSZ electrolytes, as evidenced in recent publications. However, during SOEC cell operation, the Sr ions are prone to diffuse and combine with the YSZ material (Zr) to yield SrZrO_3_. The SrZrO_3_ formed in the electrolyte is known to be an insulating phase, and its presence restricts the diffusion of oxygen ions through the solid electrolyte toward the cathode electrode. The significant hindrance to the diffusion of oxygen ions has been found to increase the polarization resistance, which influences the SOEC efficiency and device performance. Hence, to inhibit the formation of an insulating SrZrO_3_ phase in SOEC electrolytes, an interlayer was introduced containing ceria between the Sr-oxygen/cathode electrode and YSZ electrolyte [93,94]. Conclusively, the interlayers play a crucial role in SOEC systems in terms of preventing Sr diffusion, and, at the same time, provide effective pathways for oxygen ion transportation [93,94]. 

Considering the importance of the interlayer, its properties are known to play a significant role in SOEC performance. For example, a material offering an increased density of oxygen ion conduction is an ideal option for increased performance. Recently, the utilization of oxides of transition metals has been shown to improve SOEC performance by acting as a grain boundary cleaning agent and a sintering aid for ceramic electrolytes [95,96,97]. Recently, Qu et al. [98] demonstrated the integration of Fe_2_O_3_ to a samarium-doped ceria (SDC) interlayer in a SOEC system to improve the ionic conductivity of the cathode electrode. Pristine and Fe_2_O_3_ doped SDC were synthesized using a glycine-nitrate method, whereas the YSZ electrolyte and (La_0.6_Sr_0.4_)_0.95_Co_0.2_Fe_0.8_O_3_-_δ_ (LSCF) were commercially acquired; the latter was used as the oxygen electrode. For the hydrogen electrode, a glycine-nitrate method was used to obtain NiO. The doped and undoped SDC were prepared by pressing the relevant powders into green sheets (1250 °C for 4 h at a pressure of 10 MPa). During symmetrical cell testing conducted at 800 °C, a significant advantage of Fe_2_O_3_ doping was observed in terms of oxygen electrode polarization resistance and electrolysis current. The electrolysis current measured at 1.5 V during the symmetrical cell testing revealed a current density of 0.3 A cm^−2^ for the undoped system, which was found to be increased to 0.5 A following Fe_2_O_3_ doping. On the other hand, the oxygen electrode polarization resistance of undoped systems was noted to be 0.22 Ω cm^−2^, showing a significant decrease to 0.09 Ω cm^−2^ in the presence of the Fe_2_O_3_ dopant. Importantly, the introduction of a Fe_2_O_3_ interlayer dopant led to improved ionic conductivity, cathode electrode stability, and hydrogen production (calculated to be 132 mL cm^−2^ h^−1^ when undoped vs. 195 mL cm^−2^ h^−1^ when doped), which, in turn, boosted the efficiency of SOEC.

## 5. Potential Application in Commercial Electrolyzers

The commercial applicability of the catalyst in large-scale electrolysis is the most important aspect of the hydrogen electrolysis industry. Being a cheap, stable, and abundant material, Fe_2_O_3_ could be a potential candidate as a catalyst for the electrolysis industry in the future. In recent research, the applicability of Fe_2_O_3_ has been successfully tested in AWE, AEMWE, and SOEC systems. Specifically, in basic electrolytes and high-temperature ceramic systems, Fe_2_O_3_ showed significant stability. However, its poor stability in low pH conditions restricts its use in PEM-based electrolyzers. Owing to its intrinsic and extrinsic characteristics, Fe_2_O_3_ is mainly used in OER applications and bifunctional systems. Its utilization as solely a HER catalyst has not been widely studied. As discussed above, the direct utilization of Fe_2_O_3_ in pristine form has limited applicability. However, suitable modifications and/or coupling and alloying with other materials have extended its applicability, efficiency, and stability. Based on this strategy, a wide range of catalytic systems have been proposed and tested. However, studies are limited to three- and two-electrode systems in lab-scale applications. This indicates that the testing of Fe_2_O_3_ in electrolyzer test systems is lacking, and significant effort is essential in this direction to promote Fe_2_O_3_ as a catalyst for pilot scale systems in the future. Therefore, studies directed toward the testing of Fe_2_O_3_-based catalysts in electrolyzer stacks are encouraged. Recent research and advancements have indicated that Fe_2_O_3_ could be an ideal catalyst material for the AWE, AEM, and SOEC-based electrolyzers of the future. However, the intrinsic and extrinsic characteristics of Fe_2_O_3_ are mainly exploited in OER applications and bifunctional systems following strategic modifications. Hence, significant research and development of modification strategies of Fe_2_O_3_ are essential. As presented in the discussion section, the current stages of different modifications indicate improved efficiency, selectivity, and stability of Fe_2_O_3_-based electrodes in terms of OER activity. In comparison with other metal oxides/hydroxides/alloy-based electrodes, the research focus on Fe_2_O_3_ has been limited. Hence, the coming years could provide significant milestones in Fe_2_O_3_-based OER catalysts in electrolyzer systems if suitable modification strategies are employed.

**Figure 14:**
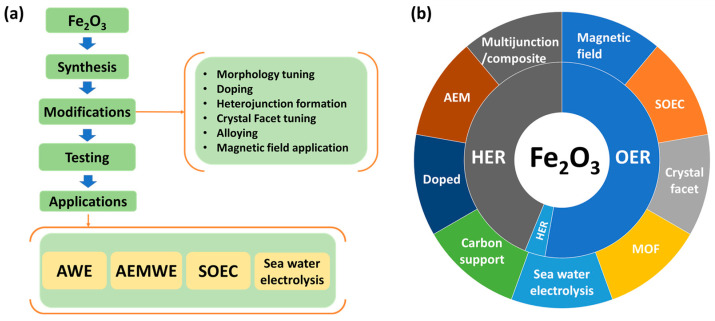
(**a**) Brief roadmap for future works on Fe_2_O_3_-based catalysts in electrolyzers. (**b**) Sunburst chart of Fe_2_O_3_ in different catalytic modes (First circle) for OER, HER, and bifunctional applications. The second circle demonstrates the different catalyst systems of Fe_2_O_3_ applications.

Based on the aforementioned discussion, we provided a brief roadmap of the use of Fe_2_O_3_ in water electrolyzers, as shown Figure 14a. The primary step involves the synthesis of Fe_2_O_3_. The synthesis protocol has to be simple and scalable industrial scale production. The next step involves the essential modifications, because the adaptation of suitable modification dictates the efficiency and stability of the catalyst. In this step, the catalyst could be modified to suit bifunctional catalysis, in addition to offering OER activity. The modification protocols need to be designed carefully to meet the scalable parameters without compromising efficiency, stability, safety, or toxicity. After the essential modifications, testing needs to be performed to assess the efficiency, stability, and necessary conditions on a lab scale. The testing step mostly determines the important parameters and conditions with which the catalyst could be utilized more efficiently. The final step involves the implementation and testing of the catalyst system in AWE, AEMWE, SOEC, and seawater electrolysis stacks to determine its potential applicability.

## 6. Discussion

Figure 14b summarizes the applications and modes of implementation of Fe_2_O_3_ in electrocatalytic water splitting systems. The low cost of Fe_2_O_3_, as well as its abundance, geographical availability, and non-toxicity, are the main aspects that drive interest in it as a catalyst in energy applications. Fe_2_O_3_ is widely employed as an OER (in addition to ORR) catalyst and as a bifunctional catalyst when combined with suitable materials or following modifications. On the other hand, its stand-alone application to HER is limited due to its poor catalysis in water reduction. Overall, the intrinsic and surface properties of Fe_2_O_3_ allow its effective implementation in water oxidation rather than water reductions. Despite widely reported OER applications, Fe_2_O_3_ still requires essential modifications to boost its OER activity and stability. As shown in Figure 14b, several strategies have been proposed to boost the OER activity and to implement it as a bifunctional/HER catalyst, as discussed below.

Crystal facet tuning and morphology: Specific facets and certain morphologies are known to offer significantly superior catalytic activities in Fe_2_O_3_ when compared to other facets and morphologies. Therefore, tuning the morphology to maximize the favorable facets via a controlled synthesis process is vital for enhanced catalytic activity, which would provide efficient charge diffusion. Research on F_2_O_3_ in this direction is limited, and thus more efforts should be made on designing synthesis schemes to obtain facets with specific morphologies.

Doping: The intrinsic characteristics of Fe_2_O_3_ are not suitable for its widespread application in, for example, water-splitting reactions. In such cases, doping could emerge as a solution to increase the abundance of catalytic sites via defects or oxygen vacancies and the density of states, which modifies the intrinsic properties of Fe_2_O_3_ to a certain extent. Even though few attempts have been reported until now, exploring a wider range of dopants could provide new insights into the properties of Fe_2_O_3_, potentially expanding its water-splitting applications.

Multijunction systems (anchoring to other materials): Anchoring Fe_2_O_3_ to different oxides, sulfides, phosphides, etc. has been undertaken extensively owing to its multiple benefits. For example, anchoring PGM catalysts to Fe_2_O_3_ could limit the excessive utilization of PGM without compromising the catalytic extent. In such cases, Fe_2_O_3_ acts as an effective platform for catalytic reaction sites. Furthermore, the presence of different metal ions (in a heterojunction system) near the Fe atoms at the surface could influence the adsorption energy and reaction intermediates which ultimately favor electrocatalytic reactions with rapid reactivity and selectivity. Alternatively, exploring other reactions for water oxidation with Fe_2_O_3_ could be beneficial in terms of achieving higher current densities with lower overpotentials. Such systems are believed to represent a key strategy in fabricating membrane-less or decoupled electrolysis systems. Moreover, heterojunctions having an effective interface with a tuned electronic structure are beneficial for the electron/hole transfer process.

Carbon material supports: The limited charge conductivity of Fe_2_O_3_ could be overcome by providing a support material for improved electrocatalytic activity. Specifically, limiting the nanostructure of Fe_2_O_3_ to one or two dimensions and providing a highly conductive platform such as graphene (including other carbon supports) could boost the catalytic activity. Carbon-supported Fe_2_O_3_ not only overcomes the limited conductivity but also improves the penetration of the electrolyte, increases back contact, increases the number of reaction sites, expands the effective interface, and provides a solid foundation for charge collection and diffusion/transfer.

Fe_2_O_3_-MOF: By utilizing a controlled and systematic synthesis (precursors) approach, a wide range of Fe_2_O_3_-MOFs have been obtained, offering tunable porosity and morphology and increased active surface area, resulting in excellent OER and HER activities. However, very limited literature in this direction limits our understanding, and thus, more mechanistic insights are needed.

Seawater electrolysis: Seawater electrolysis is an ambitious target in electrolysis, as sources of freshwater are limited and are essential to human needs; thus, the utilization of seawater and domestic sewage water is encouraged. Such an implementation could also offer benefits in terms of cost, efficiency, and availability. Fe_2_O_3_ has shown a certain degree of water-splitting activity with significant stability and selectivity in some studies. Specifically, the Fe_2_O_3_ has been shown to withstand unwanted and interfering ions, which otherwise decrease conductivity and negatively affect the catalytic properties. In some experiments, Fe_2_O_3_ (anchored system) performed significantly better than commercial RuO_2_ OER and Pt/C HER catalysts, indicating exceptional selectivity, stability, and efficiency in seawater electrolysis. A more detailed mechanistic study in this direction could potentially provide insights into Fe_2_O_3_ charge dynamics which, in turn, would benefit the development of highly efficient catalysts for seawater splitting in the future.

Fe_2_O_3_ under an applied magnetic field: Magnetic fields applied during electrolysis improve the catalysis by accelerating the OER and HER activities. Specifically, a magnetic field applied to γ-Fe_2_O_3_ alters the surface super-hydrophilic property that ensures the release of O_2_ bubbles at the surface, along with efficient penetration of electrolyte. Additionally, the magnetic field provides an added Lorentz and Kelvin force for the removal of O_2_ bubbles that ultimately results in improved water-splitting activity.

Fe_2_O_3_ in AEMWE: The cracking of catalysts in AEMWE affect their long-term activity and stability. This problem could be solved by using Fe_2_O_3_ (with Co) catalyst that inhibit cracking. Hence, Fe_2_O_3_ may play a crucial role in AEMWE in membrane-based electrolyzers in the future. Significant research in this direction is essential, as it could benefit fabrication of cost-effective electrolysis systems.

Fe_2_O_3_ in SOEC: The integration of Fe_2_O_3_ to a samarium-doped ceria (SDC) interlayer in SOEC systems improves the ionic conductivity of the cathode electrode. The introduction of Fe_2_O_3_ as an interlayer dopant improves the cathode electrode stability and hydrogen production. More dedicated research in this direction could provide new insights regarding the applicability of Fe_2_O_3_ in high-temperature electrolysis.

## 7. Summary and Outlook

The discussion presented in this review offers an important and underlying strategy regarding the use of Fe_2_O_3_ in electrocatalytic water splitting systems. Among the explored metal oxides, hematite provides the most cost-effective and efficient option for electrolysis applications. The abundance, non-toxicity, cost, and stability of basic electrolytes are the main advantages of Fe_2_O_3_ electrodes compared to other oxide counterparts. Thus, Fe_2_O_3_ offers enormous potential in future energy devices due to its versatile applicability. However, the intrinsic properties of Fe_2_O_3_ limit its application as an efficient catalyst. Despite its wider pertinence in photoelectrochemical water splitting applications, its implementation in electrolysis is limited; it is nonetheless a promising option, and thus, novel strategies in this direction are essential.

In electrolysis, Fe_2_O_3_ is widely used as an OER catalyst, in addition to bifunctional and HER catalysts, when combined with suitable materials and following the application of suitable modifications. The most common modification strategies applied to Fe_2_O_3_ include crystal facet and morphology tuning, doping, the creation of multijunction systems (anchoring with other materials), use with carbon supports, MOF linking, seawater electrolysis, the use of the magnetic field effect, and the application of AEMWE and SOEC systems. The various modifications implemented with Fe_2_O_3_ offer a wide range of advantages that benefit its OER, HER, and/or bifunctional characteristics. In light of this, the discussion presented here provides a comprehensive summary of advancements regarding the use of Fe_2_O_3_ catalysts with different modifications. Each modification provides unique changes and improvements in Fe_2_O_3_ to improve its capabilities. For example, Fe_2_O_3_ combined with Fe_2_Se_3_ or Ni_3_S_2_/N-doped carbon NiO shows the lowest overpotential (+160 and +188 mV) ever reported for the Fe_2_O_3_ in 1 M KOH, generating an OER current density of 20 and 52 mA cm^−2^, whereas Co-Fe_2_O_3_ shows exceptional stability of over 500 h when utilized in AEMWE systems. Despite its excellent performance in water splitting, reports addressing its use in electrolysis are limited when compared with those for other oxides. Therefore, significant research is essential to realize the full potential of Fe_2_O_3_ as a cost-effective and efficient water-splitting catalyst. Until now, the main focus has been in the direction of multijunction systems, whereas the doping strategy, facet tuning, and magnetic field effects have been rarely discussed. Therefore, a more significant focus in these directions could open up further potential of Fe_2_O_3_ as an efficient water-splitting catalyst. The thorough discussion on Fe_2_O_3_ presented here offers a solid platform for future research regarding the extensive modifications required to develop a highly efficient, stable, and cheap catalyst for electrocatalytic OER and HER. Importantly, the valuable insights presented here will offer detailed guidelines for the efficient design of Fe_2_O_3_-based catalysis systems.

## Figures and Tables

**Table 1:** Fe_2_O_3_ electrodes employed in electrolysis and their experimental and electrochemical properties.

Catalyst/Electrode	Synthesis Method	Electrolyte	Stability	Overpotential mV vs. SHE	Tafel Slope (mV dec^−1^)	Ref
Ni and Zn-doped Fe_2_O_3_	Combustion method	0.1 M KOH	-	OER: 350 (for 10 mA cm^−2^)	OER: 26	[40]
Se-Fe_2_O_3_@Ni/NiO	Thermal method	1 M KOH	21 h	OER: 205 (for 10 mA cm^−2^)	OER: 36	[41]
P-doped Fe_2_O_3_/ZnO	Chemical bath deposition/hydrothermal/CVD	1 M KOH	24 h	OER: 250 HER: 139 (for 10 mA cm^−2^)	OER:40 HER: 66	[42]
Zn and S co-doped Fe_2_O_3_	Hydrothermal/CVD	1 M KOH	100 h	OER: 350 (for 500 mA cm^−2^)	OER: 47.5	[43]
Co_3_O_4_/Fe_3_O_4_	Co-precipitation method	0.1 M KOH	8 h	OER: - HER: -	OER: 62 HER: 102	[44]
C-doped CoFe_2_O_4_/Fe_2_O_3_	Calcination approach	1 M KOH	5.5 h	OER: 260 HER: 236 (for 20 mA cm^−2^)	OER: 183 HER: 146	[45]
CoMo/Fe_2_O_3_	Hydrothermal/Electrodeposition methods	1 M KOH	100 h	OER: 266 HER: 71 (for 10 mA cm^−2^)	OER: 54 HER: 85	[46]
Fe_2_O_3_@CuO	Hydrothermal	1 M KOH	25 h	OER: 230 HER: 130 (for 10 mA cm^−2^)	OER: 54 HER: 77	[47]
IrO_2_–Fe_2_O_3_	Thermal decomposition	0.5 M H_2_SO_4_	600 cycles	HER: 78 (for 10 mA cm^−2^)	HER: 36.2	[48]
RuO_2_–Fe_2_O_3_	Synthesis thermal treatment	1 M KOH	-	OER: 292 (for 500 mA cm^−2^)	OER: 56.08 HER: −43	[49]
RuO_2_/Fe_2_O_3_	Synthesis	1 M KOH	18 h	OER: 386 HER: −239 (for 10 mA cm^−2^)	OER: 67 HER: 97	[50]
Fe_2_O_3_@NiO	Hydrothermal	1 M KOH	20 h	OER: 224 HER: 187 (for 10 mA cm^−2^)	OER: 20 HER: 53.8	[51]
WO_3_/Fe_2_O_3_-NiO	Chemical etching reaction and decomposition	1 M KOH	100 h	OER: 211 (for 100 mA cm^−2^)	OER: 39.5	[52]
Fe_2_O_3_/Ni(OH)_2_	Electrodeposition	1 M KOH	18 h	OER: 291 (for 10 mA cm^−2^)	OER: 53.7	[53]
Ni_1_Fe_2_@Fe_2_O_3_@C	High-temperature calcination	1 M KOH	30 h	OER: 271 (for 10 mA cm^−2^)	OER: 78	[54]
Fe_2_O_3_-MnO	Sol-gel method	1 M KOH	1000 cycles	OER: 370 (for 10 mA cm^−2^)	OER: 66	[55]
Fe_2_O_3_ǁǁFeP	Electrochemical oxidation/solution self-assembly/pyrolysis	1 M KOH	20 h	Hydrazine overpotential: 0.61 V HER: 138 (for 10 mA cm^−2^)	OER: 179.2 HER: 63.9	[56]
Fe_2_O_3_/FeP	Hydrothermal	1 M KOH	12 h	OER: 264 (for 10 mA cm^−2^)	OER: 47	[57]
Fe_2_O_3_/FeS	Hydrothermal	1 M KOH	10 h	OER: 264 (for 40 mA cm^−2^)	OER: 90	[58]
FeS/Fe_2_O_3_	Chemical etching/solvothermal	1 M KOH	10 h	OER: 266.5 (for 10 mA cm^−2^)	OER: 51.17	[59]
Fe_2_Se_3_/Fe_2_O_3_	Synthesis	1 M KOH	24 h	OER: 160 (for 20 mA cm^−2^)	OER: 30.02	[60]
Fe_2_O_3_/CNT	Co-precipitation method	1 M KOH	12h	OER: 383 (for 10 mA cm^−2^)	OER: 62	[61]
Fe/Fe_2_O_3_-Fe-N-doped C	Pyrolysis	0.1 M KOH	10h	OER: 0.69 V vs Ag/AgCl (for 10 mA cm^−2^)	OER:77.5	[62]
Fe_2_O_3_/g-C_3_N_4_	Thermal method	0.5 M KOH	10 min	OER: 425 (for 10 mA cm^−2^)	OER: 280	[63]
Fe_2_O_3_-C	Pyrolysis	1M KOH	48 h	HER: 245 (for 10 mA cm^−2^)	HER: 76.6	[64]
Ni_3_S_2_/Fe_2_O_3_/N-doped carbon	Thermal process/CVD	1 M KOH	200 h	OER: 188 (52 mA cm^−2^) HER: 78 (10 mA cm^−2^)	OER: 64.3 HER: 115.8	[65]
Fe_2_O_3_/N-graphene	Co-deposition (hydrothermal and electrodeposition)	1 M KOH	22 h	OER: 313 (for 100 mA cm^−2^)	OER: 81	[66]
Fe_2_O_3_/MWCNT	Pulsed laser ablation	1M KOH	10 h	OER: 310 (for 10 mA cm^−2^)	OER: 20.35	[67]
MOF-Fe_2_O_3_	Solvothermal	1 M KOH	20 h	OER: 439 HER: 230 (for 10 mA cm^−2^)	OER: 99 HER: 100	[68]
Fe_2_O_3_/NiO	Chemical bath deposition	1 M KOH	50 h	OER: 182 (for 10 mA cm^−2^)	OER: 45	
		Sea water		OER: 291 (for 1 A cm^−2^)		[69]
		Domestic sewage		OER: 329 (for 1 A cm^−2^)		
P-Fe_2_O_3_-CoP	Hydrothermal-gas-phase phosphorization process	Freshwater	12 h	OER: 250 HER: 219 (for 10 mA cm^−2^)	OER: 42 HER: 79	[70]
		Seawater		OER: 270 HER: 152 (for 10 mA cm^−2^)	OER: 59 HER: 95	
RuNi-Fe_2_O_3_/IF	Hydrothermal	1 M KOH	100 h	OER: 329 HER: 75 (for 100 mA cm^−2^)	OER: 60.85 HER: 85.08	[71]
		1 M KOH + seawater		OER: 424 HER: 298 (for 100 mA cm^−2^)	OER: 69.58 HER: 114.31	
Co-Fe_2_O_3_ (AEM)	Microwave-assisted hydrothermal	1.0 M KOH	500 h	OER: 310 (for 10 mA cm^−2^)	OER: 29	[72]

## Data Availability

Data are contained within the article.
